# Ultrasonic irradiation in the synthesis of nanohydroxyapatite: a chemically friendly technique for improving hemocompatibility and antibiofilm applications

**DOI:** 10.3762/bjnano.17.68

**Published:** 2026-07-29

**Authors:** Juan Mendoza Turmero, Cristina Parra Pantoja, Marcos Sabino Gutiérrez, Milagro Fernández-Delgado, Claudia Alvarado-Castillo, Damarys Soto Gil, María E Gomes Gomes, Yony Gutiérrez Barrios, Daniel Suárez Arteaga

**Affiliations:** 1 Department of Materials Science, Simón Bolívar University, Caracas, Venezuela; 2 Functional Nanostructured Materials Laboratory, Center for Materials Engineering and Nanotechnology, Venezuelan Institute for Scientific Research (IVIC). Miranda State, Venezuela; 3 Department of Chemistry, B5ida Group, Simón Bolívar University, Caracas, Venezuela; 4 Bionanotechnology Laboratory, Center for Materials Engineering and Nanotechnology, IVIC. Miranda State, Venezuela; 5 Laboratory of Hemostasis and Vascular Genetics, Center for Biophysics and Biochemistry, IVIC. Miranda State, Venezuela; 6 Nanomaterials Characterization Laboratory, Center for Materials Engineering and Nanotechnology, IVIC. Miranda State, Venezuela

**Keywords:** antibiofilm properties, hemocompatibility, nanohydroxyapatite, ultrasonic irradiation

## Abstract

This study explores the synthesis of nanohydroxyapatite (nHA) using high-frequency ultrasonic irradiation (UI), a chemically friendly technique aligned with green chemistry principles. Synthesis was achieved by varying the UI time (15, 30, 45, and 60 min) and the reaction medium. Water-based mixtures were used, that is, water/acetone (W/ACET), water/tetrahydrofuran (W/THF), and water/ethanol (W/ETOH). The resulting nHA was characterized structurally using FTIR, XRD, TGA, FESEM, and specific surface area determination. These analyses revealed the formation of carbonated nHA similar to biological apatite, with distinct morphologies and particle sizes dependent on the solvent system used. All synthesized materials exhibited high thermal stability and yields exceeding 80%. Hemocompatibility studies showed that nHA samples obtained in the W/THF mixture presented very low hemotoxicity (hemolysis < 2%), and did not affect platelet ADP aggregation processes. Microbiological assays showed a significant reduction in *Pseudomonas aeruginosa* biomass production (*p* < 0.05) after 24 h, particularly for nHA synthesized with the W/ETOH mixture. There was also a less bacterial colonization, cellular aggregates, or formation of specialized structures after treatment with this bioceramic, compared to the commercial hydroxyapatite, suggesting potential antibiofilm properties. This research presents an original and efficient method for producing nHA as a promising biomaterial for tissue engineering.

## Introduction

**Figure 1:**
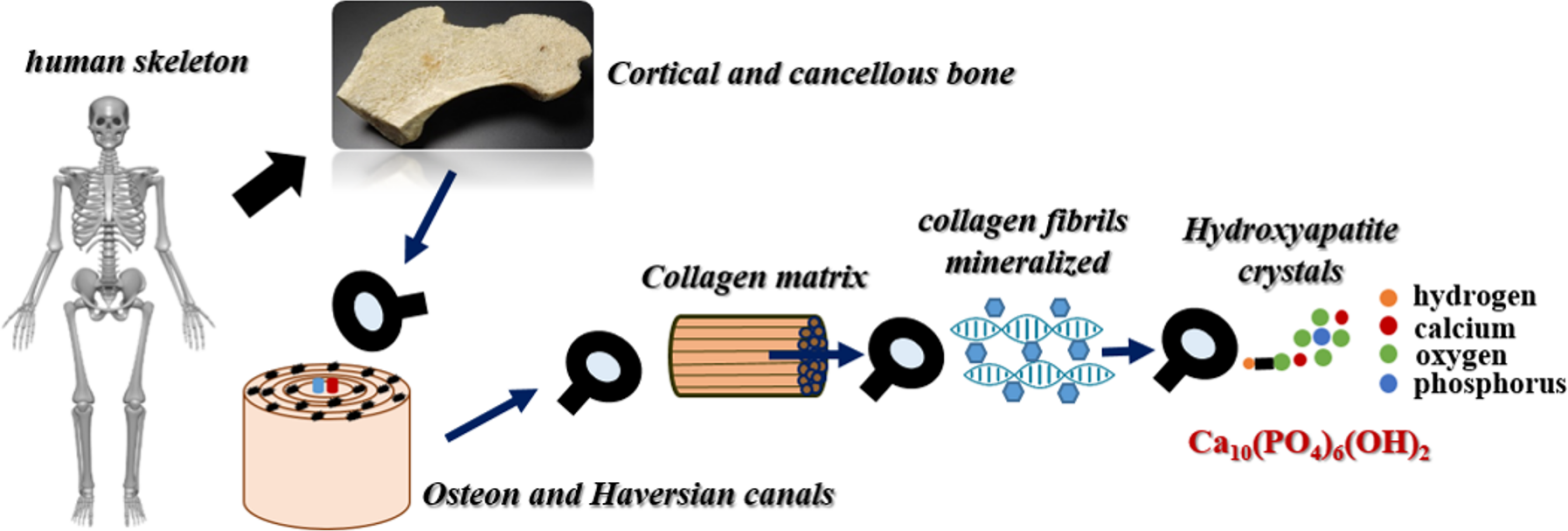
The hierarchical structure of the human skeletal system. The images “human skeleton” and “cortical and cancellous bone” have been reproduced from the links: https://pixabay.com/es/illustrations/esqueleto-humano-huesos-cr%c3%a1neo-1158318/ and https://pixabay.com/es/photos/el-detalle-de-los-huesos-4451356/ (© 2019). This content is not subject to CC BY 4.0.

Nowadays, there is a high demand for the development of bone substitutes that are biocompatible, bioactive, mechanically strong, and well tolerated by the immune system [1], which represents a major challenge for science. Prostheses used to repair skeletal system defects and diseases should be customized devices to respond specifically to physiological demands of each different organism [2–4]. For this reason, most recent research shows a trend toward biomimicry or the use of materials with structures and properties very similar to those of biological origin [5]. This process involves reaching nanodimensions and reproducing the monolithic structure of bone [6], which has been difficult to achieve until now. Bones are a complex system, composed of hydroxyapatite crystals in the form of nanoneedles, that are closely bound together with type-I collagen fibrils [7], as shown in Figure 1.

**Figure 2:**
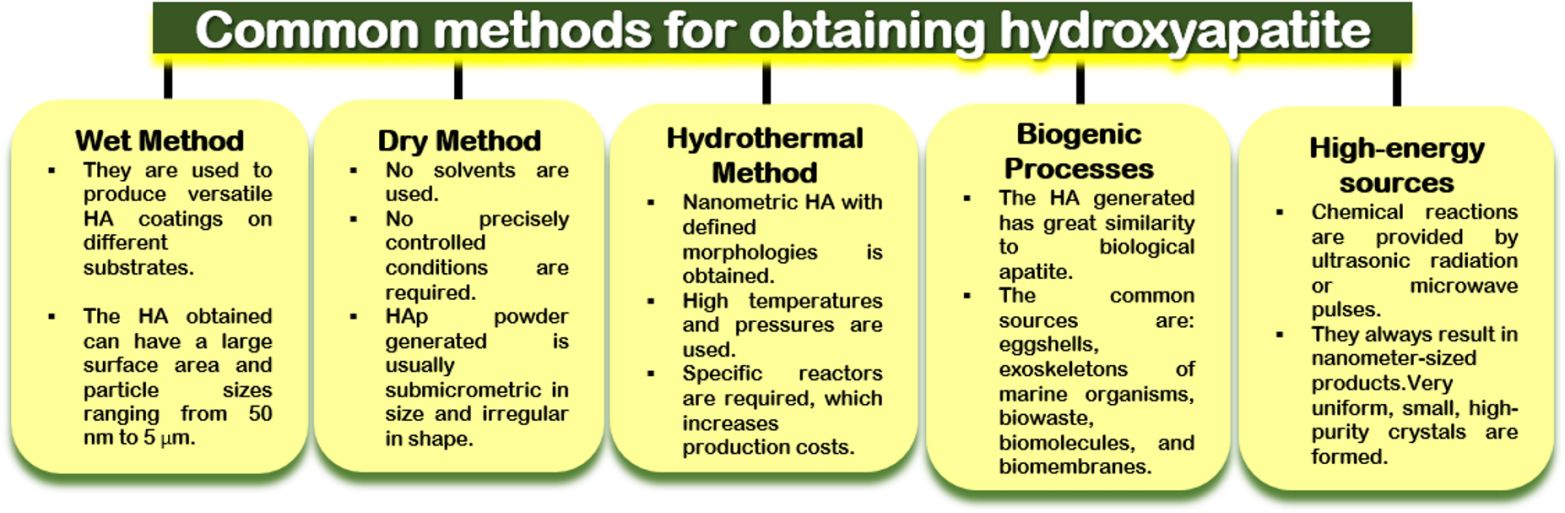
Advantages and disadvantages of the main methods for obtaining hydroxyapatite.

Hydroxyapatite crystals are an important source of calcium and phosphorus, which give bone its density, mechanical strength, and bioactivity [8]. Methods for obtaining nanohydroxyapatite (nHA) can be grouped into five categories, namely, (i) wet methods, (ii) dry methods, (iii) high-temperature processes, (iv) from biogenic sources, and (v) with high-energy sources [9–10]. The resulting hydroxyapatite is used for bone scaffolds and fillers [11], devices for controlled drug release [12], and implant coatings [13]. The most widely reported method for obtaining hydroxyapatite is the hydrothermal method [14]. However, this synthesis procedure involves high energy consumption and prolonged reaction times [15]. Figure 2 summarizes some of the advantages and disadvantages of each synthesis method.

To propose alternative methodologies to those mentioned in Figure 2, we implemented techniques with low energy consumption that allow for ease in handling reaction variables. One novel and versatile process for obtaining hydroxyapatite is sonochemistry, which uses ultrasound as energy source [16]. Sonochemistry shows great potential and has therefore aroused significant interest. This is because the chemical effects of ultrasound do not originate from a direct interaction between sound waves and the molecular species present in the medium. Rather, they derive mainly from acoustic cavitation, a physical phenomenon that efficiently concentrates diffuse sound energy and is much more powerful than common thermal energy transfer.

When high-frequency sound waves pass through a liquid medium, a large number of microbubbles form, grow, and collapse within intervals as short as a few microseconds. According to sonochemical theory calculations and experimental evidence, this process can generate local temperatures as high as 3000 K and local pressures as high as 1000 atm, heating and cooling the medium at rates as high as 10^9^ K/s [16]. In this harsh reaction environment, ions, free radicals, and other highly reactive chemical species are easily generated without the need for reaction initiators. For this reason, ultrasonic irradiation is widely used in the formation, grinding, dispersion, and activation of many inorganic compounds at the micro- and the nanoscale [17–18].

Previous research has reported the production of carbonated hydroxyapatite, which has a structure very similar to that of biological apatite, using high-frequency ultrasound [19]. Due to the complexity of the physicochemical processes that occur in the medium during sonochemical synthesis, the resulting particles can exhibit great variability in morphology. These particles can be in the form of needles, regular and irregular spheres, rods, and many others [20–21]. They also exhibit high degrees of crystallinity, high purity, and nanoscale dimensions [22]. The fascinating properties of the resulting materials have led to very promising results, further diversifying its multiple applications [23]. Some studies have also shown that synthesis parameters such as the type of solvent, ultrasonic irradiation time, and the nature of the reactants used as nHA precursors, directly influence the microstructure of the resulting material [24]. Due to the simplicity of the reaction medium, low energy consumption, and short processing times required to obtain the products, nHA synthesis carried out with ultrasonic irradiation complies with the precepts of green chemistry. Specifically, this method operates at low temperatures and atmospheric pressure, significantly reducing energy consumption compared to traditional hydrothermal routes that require more drastic conditions. In addition, selecting aqueous solvents contributes to a greener process, aligning with the goal of reducing chemical toxicity through proper solvent selection and dilution [25]. Green chemistry involves designing of chemical processes and products that reduce or eliminate the use and generation of hazardous substances, through a sustainable approach, that meets current needs without compromising the availability of resources and the quality of life of future generations [26]. Nanohydroxyapatite is distinguished by its remarkable osteoinduction and osteoconduction capabilities, making it a bioceramic with great potential in biomedical engineering. Its low hemotoxicity [27–28] and antibacterial properties [29–30] further expand its applicability in the field of biomaterials. It is also important to determine the antibiofilm effectiveness of this bioceramic against opportunistic bacteria such as *Pseudomonas aeruginosa*. This pathogen is responsible for high rates of morbidity, mortality, as well as multidrug resistance, worldwide [31]. A key factor in its pathogenicity and transmission is its ability to form virulent biofilms, which are particularly difficult to eradicate. Biofilms are communities of microbial cells enclosed by exopolysaccharides that adhere to inert or living surfaces. These biofilms enhance bacterial growth and survival by providing access to nutrients and protection against toxic compounds, adverse environmental conditions, antimicrobials, and the immune response of the host [32]. Biofilms are critical virulence factors for many bacterial pathogens that can cause chronic infections, accounting for more than 80% of human microbial infections. *P. aeruginosa* has been considered a model for studying nosocomial bacterial biofilms [33–34]; more effective biofilm control strategies should be developed for patients requiring indwelling medical devices.

Thanks to its multifunctionality and excellent bioactive and biocompatible properties, the synthesis, characterization, and study of hydroxyapatite continue to provide significant advantages in the development of new biomaterials. Furthermore, obtaining nHA through a green chemistry approach adds to its many existing advantages. Based on these premises, this study investigated the influence of ultrasonic irradiation on hydroxyapatite properties by varying the irradiation time and solvents used in the synthesis process. Additionally, we evaluated their hemocompatibility and ability to inhibit colonization or biofilm formation against pathogens, contributing to the exploration of new nHA-based biomaterials for use in tissue engineering and bone devices.

## Results and Discussion

### Infrared study

**Figure 3:**
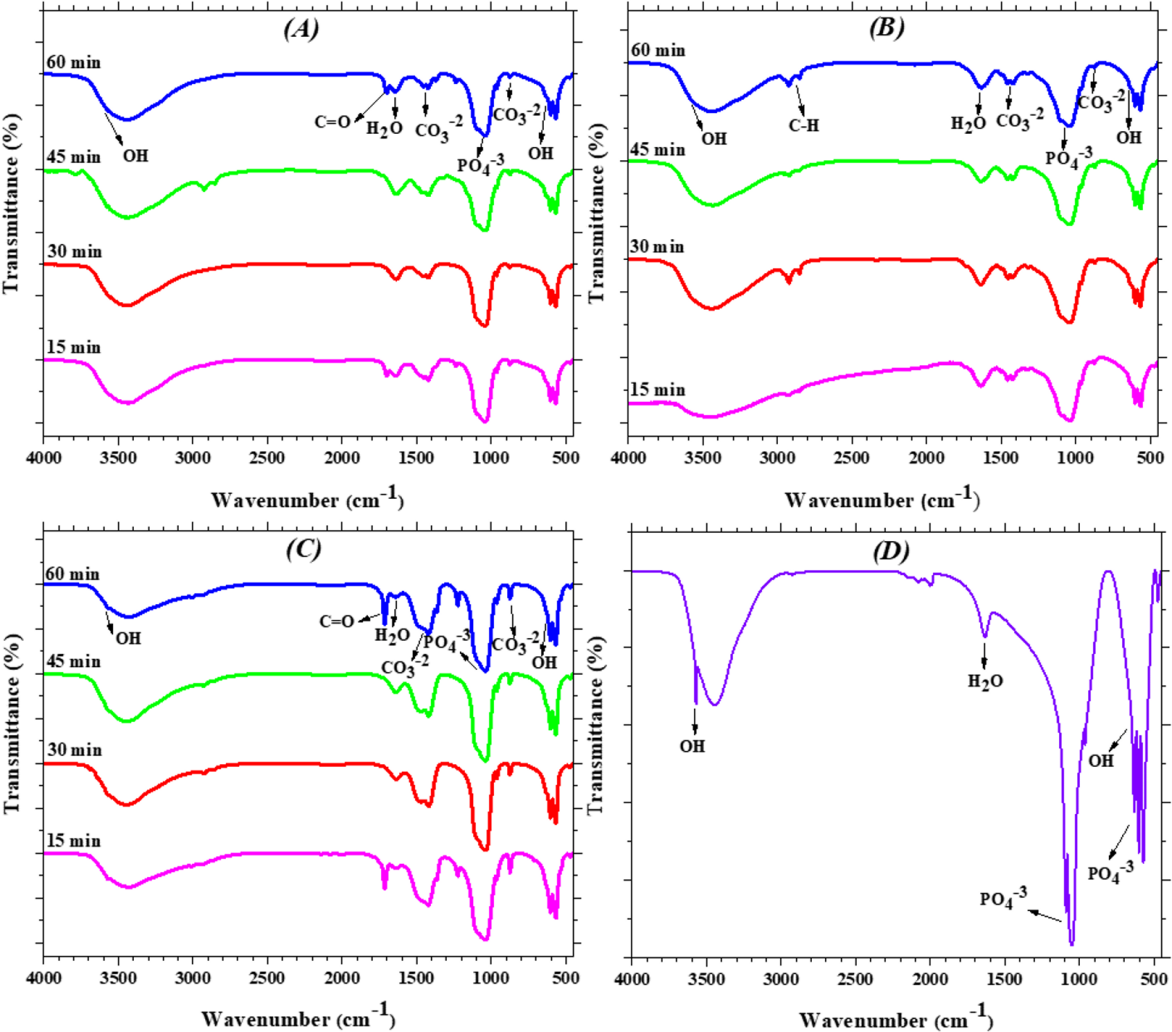
FTIR spectra of nHA obtained in: (A) W/ACET, (B) W/THF, and (C) W/ETOH using 15, 30, 45, and 60 min of UI; (D) FTIR spectrum of commercial hydroxyapatite.

Figure 3 shows the FTIR spectra of hydroxyapatite prepared using mixtures of water/acetone (W/ACET), water/THF (W/THF), and water/ethanol (W/ETOH) with varying ultrasonic irradiation (UI) times between 15 and 60 min. The spectra in Figure 3A–C show the characteristic absorption bands of the hydroxyapatite structure [35–36]: the presence of OH groups in the ranges of 3573–3558 and 633–628 cm^−1^ is verified, as are the vibrational modes of PO_4_^3−^ ions, that is, ν_1_ at 963–962 cm^−1^, ν_2_ at 476–471 cm^−1^, ν_3_ at 1097–1038 cm^−1^, and ν_4_ at 603–656 cm^−1^. Furthermore, no bands corresponding to organic solvent residues (such as ethanol, acetone, or THF) were detected, confirming the efficacy of the exhaustive washing process. However, it is important to note that while FTIR confirms the absence of major organic impurities, the inherent sensitivity of this technique might not be sufficient to detect ultratrace amounts of residual solvents adsorbed on the surface or trapped within the nanostructured hydroxyapatite matrix.

The remaining bands in the spectra shown in the spectra in Figure 3 indicate the presence of carbonate groups within the wave number ranges of 875–873 cm^−1^ (ν_2_) and 1499–1420 cm^−1^ (ν_3_). This certifies that carbonated hydroxyapatite was obtained under all synthesis conditions [37]. Additional absorption bands observed in most of the spectra in Figure 3 correspond to carbonyl group vibrations between 1739 and 1701 cm^−1^ and to adsorbed water molecules at 1636 cm^−1^ (for nHA obtained in the W/ACET system), 1634 cm^−1^ (for nHA obtained in the W/THF system), and at 1638–1634 cm^−1^ (for nHA obtained in the W/ETOH system).

Table S1 (shown in Section I, Supporting Information File 1) systematically summarizes the assignment of absorption bands for the PO_4_^3−^ and OH^−^ ions, as well as for the ν_3_ and ν_2_ vibrational modes of the CO_3_^2−^ ions. This is compared with a sample of non-carbonated commercial hydroxyapatite from Teknimed S.A. (France), which is used as a reference. The versatile structure of hydroxyapatite allows for the replacement of its constituent chemical species (calcium, phosphate, and hydroxide ions) [38–39]. This substitution process produces changes in its properties, including crystallinity, morphology, and lattice parameters [40–41]. The nHA obtained using the three aforementioned mixtures as solvents exhibited type-A, type-B, and type-AB substitutions in its structure. Type-B substitution (when carbonates occupy the PO_4_^3−^ sites) [42–43] is particularly evident through the significant shifts observed in the ν_3_ vibrational modes (doublet of the PO_4_^3−^ group) of the synthesized samples compared to the commercial hydroxyapatite sample. This change is also evident in the more pronounced width of the bands corresponding to the phosphate doublets (Figure 3A–C) when compared to the same absorption band in Figure 3D. It is important to note that the intensity of the ν_3_ band, which corresponds to type-B substitution and appears in all the spectra in Figure 3 in the range of 1424–1420 cm^−1^ is more pronounced than that of type-A substitution (1499–1493 cm^−1^) of the OH sites. This confirms that for these syntheses, the substitution effect was much greater at the phosphate sites than at the hydroxide sites. According to the literature, replacing phosphate ions is difficult because they are strongly bound to calcium cations within the hydroxyapatite structure. Replacing phosphates with other anions, such as carbonate, significantly alters the properties of this bioceramic, resulting in decreased hardness and stability [44]. However, high-frequency ultrasound provides enough energy to promote this process, which would likely be more difficult using other synthesis methods [45]. Type-A substitution (carbonate ions for hydroxide ions) was only observed in nHA obtained from W/ACET and W/THF mixtures; it was not observed in nHA synthesized using W/ETOH as a solvent. These results suggest that the free OH^−^ ions in hydroxyapatite are more strongly bound, making them very unlikely to leave it, even in a more polar medium such as that provided by the W/ETOH mixture [46]. Incorporating polyatomic anions such as carbonate into the hydroxyapatite structure creates vacancies and calcium deficiencies, which lead to the formation of a non-stoichiometric hydroxyapatite with a hexagonal structure and space group *P*6_3_/*m* [47].

Table 1 shows the Ca/P ratios determined by EDX analysis of nHA samples synthesized using 15 and 60 min of UI in W/ACET, W/THF, and W/ETOH mixtures. The nHA obtained in the W/ACET mixture was calcium-deficient at low ultrasonic irradiation times, while that obtained in W/THF was stoichiometric.

**Table 1:** Ca/P ratios determined from EDX analysis of nHA samples synthesized using 15 and 60 min of UI using W/ACET, W/THF, and W/ETOH mixtures.

Sample	Solvent	UI time (min)	Ca (wt %)	P (wt %)	Ca/P ratio

nHA	W/ACET	15	9.20	5.89	1.56
		60	12.14	6.50	1.87

	W/THF	15	18.20	11.22	1.62
		60	9.44	5.79	1.63

	W/ETOH	15	16.85	8.35	2.02
		60	3.36	1.58	2.13

The HA formed using W/ETOH as a solvent was also not stoichiometric, but unlike that prepared in W/ACET it was obtained with an excess of calcium. These results demonstrate the significant influence of the solvent type and UI time on the formation of nHA. Similar findings have been reported by other authors using different synthesis methods [48–49]. Another important result is the appearance of bands associated with the carbonyl group in some of the FTIR spectra of the synthesized nHA at certain ultrasonic irradiation times. The W/ACET and the W/ETOH mixtures (15 and 60 min of UI) and the W/THF mixture (15 and 30 min of UI) exhibited this phenomenon. This suggests that the high energy capacity of ultrasonic waves promotes in the reaction medium the formation and coexistence of another distinct mineral phase with carbonate groups in its composition. Table 2 shows the carbonate content of the nHA samples, which was calculated by integrating the absorption bands in the ν_3_ region of the CO_3_^2−^ ions in all nHA spectra in the range of 1560–1380 cm^−1^. These results indicate that the variation in the content of these ions, in relation to the solvent mixture used in the synthesis, is as follows: W/ETOH > W/ACET ≈ W/THF.

**Table 2:** Peak area determined from the FTIR spectra of nHA, for the ν_3_ vibrational modes of carbonate ions (1560–1380 cm^−1^).

Solvent	UI time (min)	Area CO_3_^2−^

W/ACET	15	15.75
	30	9.21
	45	23.28
	60	9.27

W/THF	15	15.71
	30	12.49
	45	12.54
	60	17.54

W/ETOH	15	47.92
	30	35.16
	45	34.64
	60	42.86

The spectra of the nHA samples obtained in the W/ETOH mixtures (Figure 3C) show intense and well-defined carbonate bands, particularly for the nHA prepared at 15 and 60 min of ultrasonic irradiation. This is consistent with the results shown in Table 2. Furthermore, the spectra in Figure 3A and Figure 3C show the presence of a carbonyl band at 1712 and 1726 cm^−1^, for 15 and 60 min of ultrasonic irradiation, respectively, indicating an additional contribution of carbonate groups in both spectra.

### X-ray diffraction

**Figure 4:**
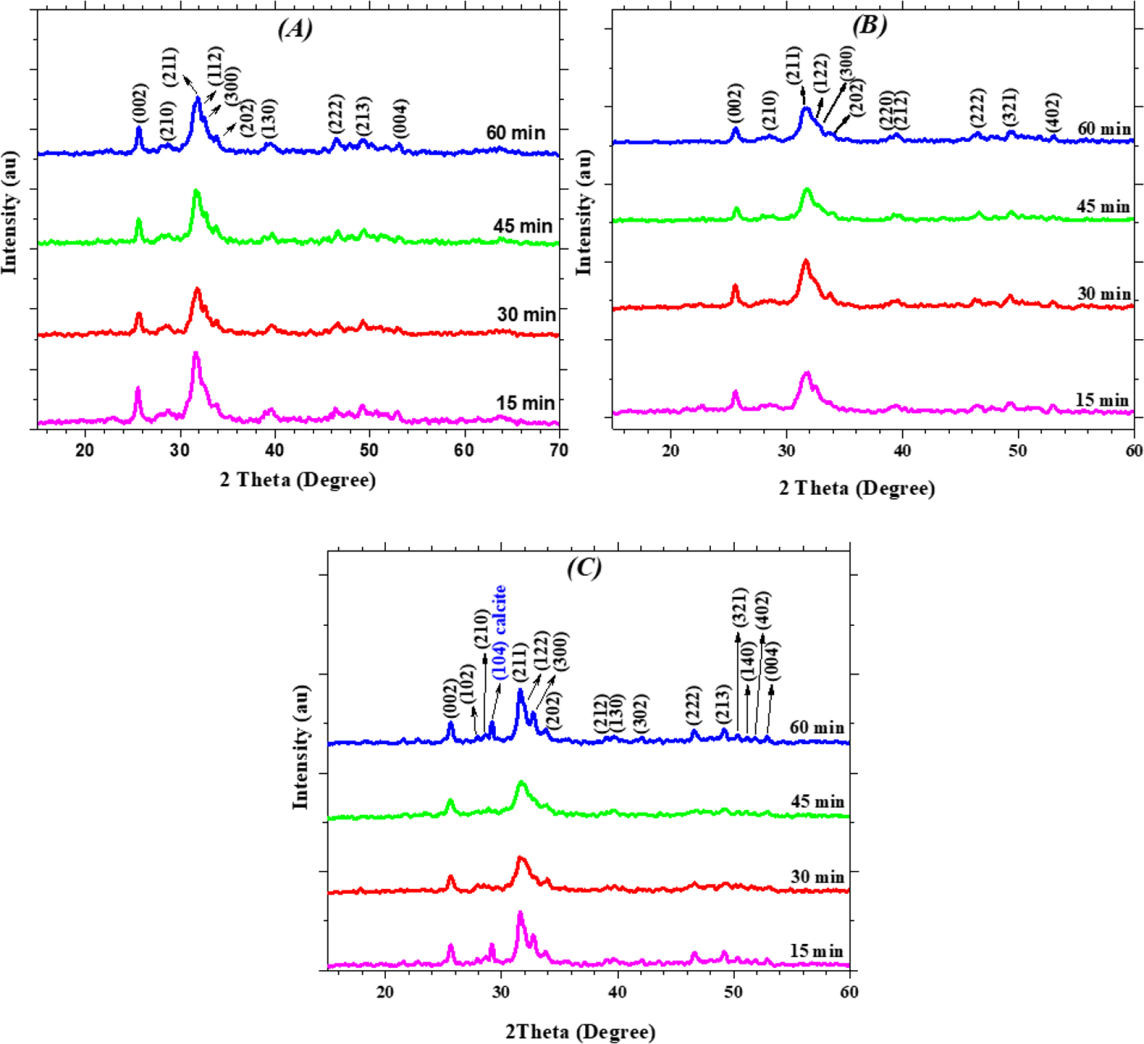
X-ray diffractograms of nHA synthesized in the mixtures: (A) W/ACET, (B) W/THF, and (C) W/ETOH; at 15, 30, 45, and 60 min of UI.

Figure 4 shows the X-ray diffractograms of the nHA obtained from the W/ACET, W/THF, and W/ETOH mixtures at various UI times. Almost all of the diffraction peaks shown in Figure 4 correspond to those reported in the literature for hydroxyapatite [50–51]. The main diffraction peak with the highest intensity appears at approximately 2θ = 31.70°, followed by three adjacent peaks at 2θ = 32.20°, 2θ = 32.90°, and 2θ = 34.22°. These results correspond to what was observed by FTIR regarding the formation of a carbonated hydroxyapatite with a hexagonal crystalline structure [52–53]. Additionally, other crystallographic planes such as (212), (130), (302), (222), (213), (321), (140), (402), and (004), are very similar to those reported for biological apatite or apatite derived from biogenic according to previous studies [54–55]. The pronounced width of all the diffraction peaks in Figure 4 indicates the nanocrystalline nature of the obtained hydroxyapatite. To verify whether the ultrasonic irradiation time induces a significant decrease in dimensions, a precise quantitative analysis of particle size was conducted using field-emission scanning electron microscopy (FESEM) and BET surface area measurements. These findings, along with their corresponding statistical evaluations, are presented and extensively discussed in section “Field emission scanning electron microscopy and particle size determination via the BET method” (see below Table 6), confirming the nanometric control achieved through the sonochemical process [56–57].

Regarding the reactions in the W/ETOH mixture, rhombohedral calcite formation (space group *R*3*c*) was confirmed by a well-defined signal at 2θ = 29.20° appearing in the diffractograms corresponding to 15 and 60 min of ultrasonic irradiation (Figure 4C). This signal corresponds to the highest-intensity calcite peak, plane (104), in accordance with reference pattern 00-002-0629 from the JCPDS database. These results were corroborated by those obtained via infrared spectroscopy, as shown in Figure 3C. The phenomenon of ultrasonic cavitation, within a polar medium such as a W/ETOH mixture, facilitates the formation of ionic species that are susceptible to rapid recombination processes, giving rise to a more stable crystalline phase.

**Figure 5:**
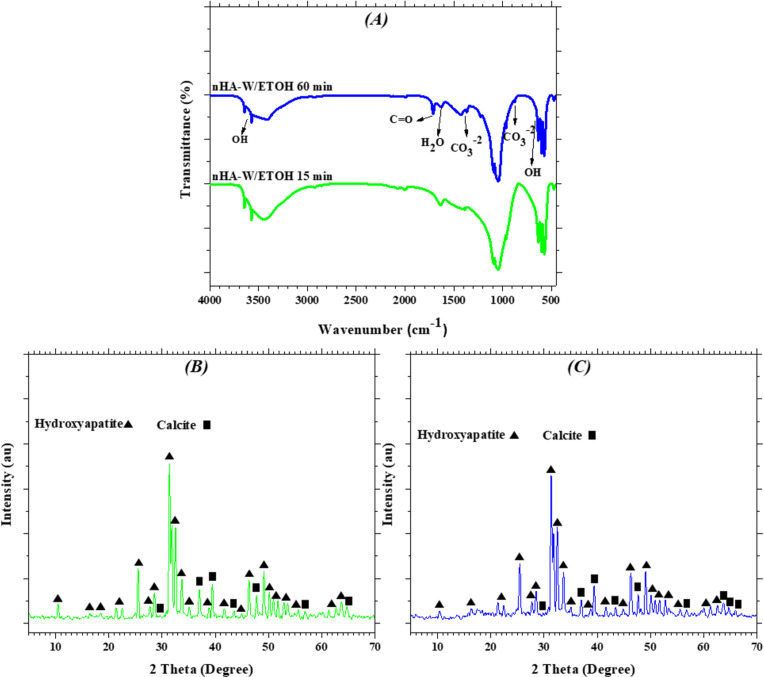
(A) FTIR spectra of the calcination residue of nHA obtained in the W/ETOH at 15 and 60 min of UI, (B) and (C) X-ray diffractograms of the calcination residue of nHA obtained at 15 and 60 min of UI, respectively.

Since all nanohydroxyapatite synthesis reactions were conducted at room temperature (approximately 25 °C) and atmospheric pressure (around 0.96 atm), values reported in the literature (Δ*G*_f_ = −12670 kJ/mol for hydroxyapatite and −1128 kJ/mol for calcite [58–59]) can be used to justify the formation dynamics of both phases. The formation of hydroxyapatite and calcite is thermodynamically favored, as indicated by their negative standard Gibbs free energy values (Δ*G*°_f_). The predominance of the hydroxyapatite phase is consistent with its higher thermodynamic stability within calcium phosphate systems. However, the appearance of calcite as a secondary phase suggests a competitive nucleation process. The high-energy environment generated by high-frequency ultrasound, coupled with the reduced dielectric constant of the W/ETOH mixture, promotes high local supersaturation levels. Under these extreme conditions, sonochemical energy facilitates the pyrolysis and oxidation of ethanol molecules at the cavitation bubble interface; this provides an alternative source of inorganic carbon for the formation of carbonate groups, even under an inert atmosphere [60]. These extraordinary conditions provide the necessary activation energy to overcome the kinetic barriers for both phases, enabling the co-precipitation of calcite alongside the more thermodynamically stable hydroxyapatite. The proportion of calcite formed increased as the ultrasonic irradiation time progressed from 15 to 60 min. This is evident from the higher carbonate content of the samples obtained at these two times (Table 2), compared to those obtained at intermediate irradiation times (30 and 45 min). To definitively confirm calcite formation in the syntheses conducted in the W/ETOH mixture, samples obtained after 15 and 60 min of ultrasonic irradiation were calcined at 1100 °C in air, and the resulting products were studied using infrared spectroscopy and X-ray diffraction. Complementary to this, a semi-quantitative phase analysis was performed on the XRD patterns to determine the relative calcite content. This estimation was based on the principle that, in a binary mixture of nanohydroxyapatite and calcite, the diffraction peak intensity of a given phase is directly proportional to its mass fraction and its reference intensity ratio (RIR) value [61]. Figure 5A shows that, after calcination, the carbonyl band disappears for the solid obtained at 15 min and the bands associated with the carbonate groups decrease significantly. In contrast, the bands of both functional groups (carbonyl C=O and carbonate CO_3_^2−^), remain in the FTIR spectrum of the sample synthesized using 60 min of UI. This behavior is associated with the formation of different proportions of calcite after 15 and 60 min of reaction (see Figure 5B,C).

These results, along with those from FTIR, demonstrate that the sonication energy was able to promote the joint precipitation of both crystalline phases in both reactions carried out at 15 and 60 min of UI. The results of the semi-quantitative phase analysis for the samples synthesized in the W/ETOH system are presented in Table 3 (information regarding the diffraction patterns, processed using a graphing and data analysis software, is available in Section II of Supporting Information File 1). This table details the mass percentages obtained for both nanohydroxyapatite and calcite after 15 and 60 min of ultrasonic irradiation, illustrating the evolution of the phase content as a function of the reaction time.

**Table 3:** Phase composition of samples synthesized in the W/EtOH system.^a^

Phase	*H*_i_	FWHM	*I*_i_	RIR	*I*_i_/RIR	nHA (%)	Calcite (%)

	nHA obtained in W/ETOH (15 min)						

nHA	119.07	0.81	102.57	1.00	102.57	93.63	–
Calcite	52.24	0.39	21.76	3.12	6.97	–	6.37

	nHA obtained in W/ETOH (60 min)						

nHA	119.07	0.81	102.57	1.00	102.57	96.79	–
Calcite	36.15	0.28	10.60	3.12	3.40	–	3.21

^a^RIR: reference intensity ratio.

These results indicate that nHA is the predominant phase (94–97%), while calcite appears as a minor secondary phase (3–6%). It is crucial to highlight that all experiments were conducted under an inert atmosphere to prevent the absorption of atmospheric CO_2_. Therefore, the presence of this minor phase is attributed to solvent sonolysis, promoted by the extreme conditions of the ultrasonic medium (*P* ≈ 1000 atm and *T* ≈ 5000 K). These conditions generate highly reactive species that lead to a specific chemical equilibrium in the W/ETOH system during the initial stages of the reaction. Furthermore, the quantification demonstrates that the nHA/calcite ratio does not show significant variations across the evaluated irradiation times (15 to 60 min). This stability confirms that the ultrasonic treatment does not promote the further growth of the secondary phase over time.

A deeper understanding of calcite formation under these conditions can be gained by considering the chemical equilibrium and the solubility of the precursor reagents. Results shown by Kitamura et al. [62] revealed that the dissolution of calcium hydroxide in the reaction medium significantly influences the nucleation of specific calcium carbonate polymorphs. In this context, the solubility product (*K*_sp_) is a key parameter for predicting the formation or dissolution of solids. For Ca(OH)_2_ in aqueous solution, this constant is 5.5 × 10^−6^ [63], which is significantly higher than the *K*_sp_ of CaCO_3_ (3.3 × 10^−9^) [63]. This marked difference explains why calcite forms preferentially at the expense of Ca(OH)_2_, as the latter is more soluble and thus more readily available in the reaction medium. Regarding the carbon source, some authors have reported that when alcohol-containing media are subjected to ultrasonic irradiation, the main decomposition products include hydrogen, methane, ethylene, aldehydes, and carbon monoxide [64]. In the present study, the W/ETOH medium provides the necessary species for calcite formation through these sonochemical pathways.

Under an inert atmosphere, the formation of calcite suggests an inorganic carbon source derived from the reaction medium itself. Through the high-frequency ultrasound, sufficient energy is available to induce ethanol pyrolysis, whose fragments interact with the hydroxyl radicals (OH^•^) generated by water sonolysis to favor the synthesis of carbonate groups. This mechanism, although complex, is supported by the efficacy of sonochemical processes in converting organic solvents into reactive species, thereby facilitating the overcoming of kinetic barriers that limit the formation of the calcite phase.

Based on the aforementioned chemical and physical factors, we propose a possible mechanistic pathway for calcite formation. While this proposal is not intended to be definitive, it aims to clarify the presence of this minor phase during the synthesis of nanohydroxyapatite in the W/ETOH system. The following sequence of chemical reactions could reasonably justify this process:


[1]

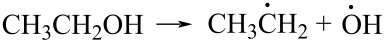




[2]

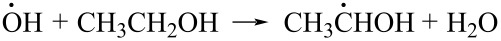




[3]

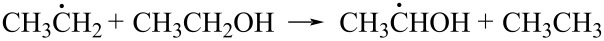




[4]

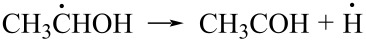




[5]

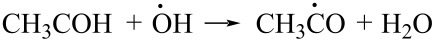




[6]

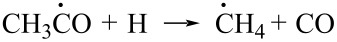




[7]

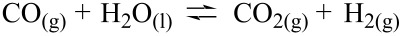




[8]

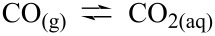




[9]

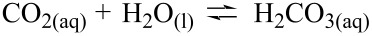




[10]

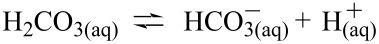




[11]

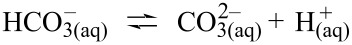



The transformation can occur very quickly due to the presence of calcium hydroxide and carbonate ions in the reaction medium:


[12]

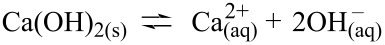




[13]

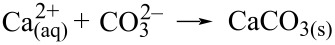



The solubility product of hydroxyapatite is a key a parameter of great interest for understanding its higher precipitation capacity compared to calcite. An acceptable value for the *K*_sp_ of HA is 2.91 × 10^−58^, as reported by L. C. Bell and coworkers [65]. According to Equation 14, this value indicates the degree to which hydroxyapatite will dissolve in water or biological fluids at standard temperature and pressure:


[14]

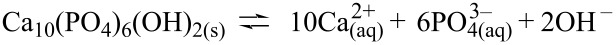



The equilibrium constant for the dissolution reaction of HA in a saturated solution is given by Equation 15:


[15] $$K_{\text{sp}}={\left[{\text{Ca}}^{2+}\right]}^{10}*{\left[{\text{PO}}_{4}^{3-}\right]}^{6}*{\left[{\text{OH}}^{-}\right]}^{2}=3.37\times 10^{-58}$$

This low solubility is vital in biological systems such as bones and teeth, where hydroxyapatite provides a stable, insoluble reserve of calcium and phosphate ions. Its use in various applications of interest to this research, such as bone grafts and coatings, is also relevant. In these applications, the slow dissolution rate is a desirable property.

### Yield in the ultrasonic synthesis of hydroxyapatite

**Figure 6:**
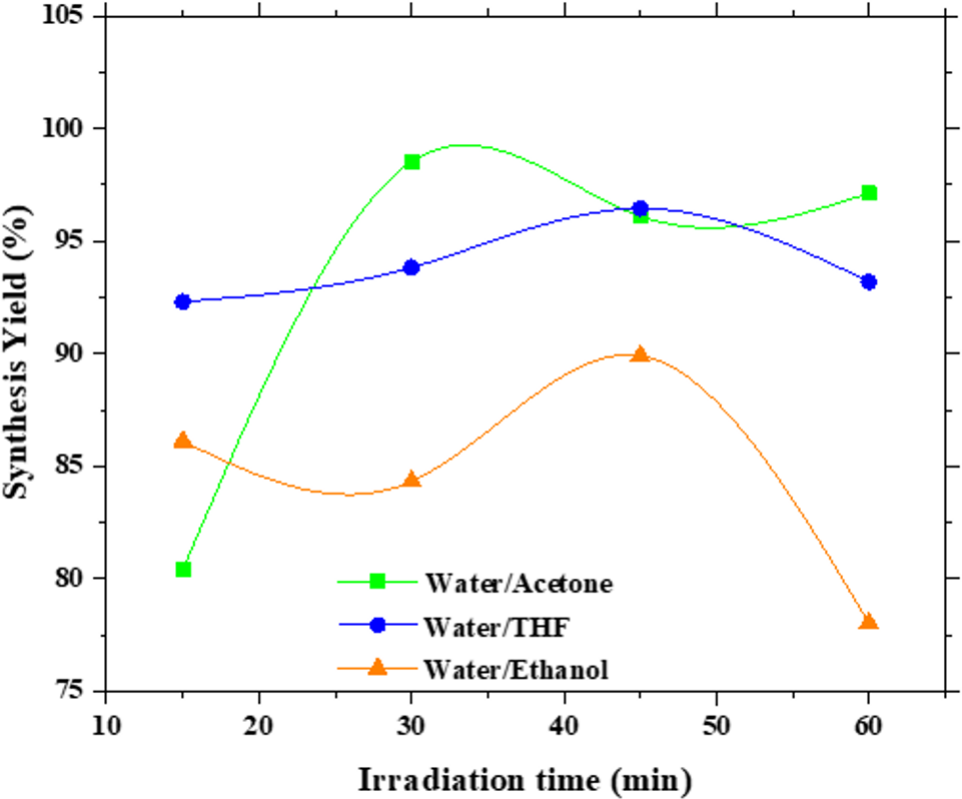
Yields obtained in the synthesis of hydroxyapatite, carried out in W/ACET, W/THF, and W/ETOH mixtures, using 15, 30, 45, and 60 min of UI.

Figure 6 shows the experimental yields obtained from the nHA synthesis using different solvent mixtures and ultrasonic irradiation times (15, 30, 45, and 60 min). The connecting lines for each of the curves serve as a guide for the eye and do not represent a specific mathematical model or kinetic fit. This visual outline highlights a consistent increase in yield, which remains steadily above 70% from the initial stages, reflecting the high efficiency of the sonochemical process under the conditions evaluated in this research. The yield values ranged from 78% to 97%; the highest yields were observed in the W/ACET mixture and the lowest were observed in the W/ETOH mixture.

The following order of effectiveness for nHA formation is established: W/ACET > W/THF > W/ETOH. This trend can be attributed to the dielectric constants [66], water solubility, and dipole moments of the solvent mixtures as shown in Table 4.

**Table 4:** Some physical constants of interest for water, THF, ethanol, and acetone.^a^

Mixture/substance	Dielectric constant, ε	Dipole moment, µ (D)	Solubility

water/acetone	50.40	–	–
water/THF	44.29	–	–
water/ethanol	52.75	–	–
water	80.10	1.82	–
THF	7.58	1.75	in water (s)
acetone	20.70	2.85	in water (∞)
ethanol	24.50	1.69	in water (∞)

^a^µ: dipole moment; (s): soluble; (∞): infinitely soluble; ε_mixture_ = 0.5(ε_H₂O_ + ε_solvent_).

The W/ACET mixture was the most effective at promoting separation, solvation, and stabilization of Ca^+2^, OH^−^, and PO_4_^3−^ ions. This resulted in a higher recombination rate and the rapid formation of hydroxyapatite and, consequently, higher yields. In contrast, the W/THF mixture, despite yield values above 90%, showed a decrease in synthesis yield. This is due to a notable drop in the mixture’s dielectric constant and the dipole moment of THF, which reduced the nucleation rate of hydroxyapatite. A significant increase in polarity did not result in higher yields for the W/ETOH mixture. This is because the W/ETOH system has unique hydrogen bond interactions that limit the availability of active sites for the solvation of calcium, phosphate, and hydroxide ions, which resulted in a notable decrease in the production yield of nHA. Other authors have reported similar results in the sol–gel synthesis of nHA. These results, however, were not obtained with solvent mixtures. They provide clear evidence of the marked influence of the reaction medium on the production of hydroxyapatite in the presence of alcohols, ketones, and aprotic solvents such as tetrahydrofuran [67].

Regarding the reaction mechanism, while the fundamental stoichiometric chemical pathway leading to hydroxyapatite precipitation remains identical across all systems, a detailed step-by-step molecular mechanism for each medium is not proposed since the organic co-solvents do not alter the primary chemical nature of the reacting species. Instead, the solvent media (water/ethanol, water/THF, and water/acetone) act as physicochemical modulators of the overall reaction kinetics and cavitation behavior, which directly explains the observed discrepancies in both yield and nanostructure dimensions. The introduction of these organic co-solvents significantly alters the dielectric constant of the medium, modifying the solubility and supersaturation kinetics of calcium and phosphate ions. Furthermore, the higher vapor pressures of volatile solvents like THF and acetone compared to ethanol can lead to a “cushioning effect” inside the cavitation bubbles. This dampens the local temperatures and pressures generated during bubble collapse, consequently affecting the reaction kinetics and the ultimate synthesis yield. Additionally, the specific coordinating capacity of THF and acetone toward Ca^+2^ ions, along with their differential adsorption on specific crystal facets, plays a decisive role in governing the nucleation-to-growth ratio.

The ultrasonic irradiation time required to obtain these high yields was highly variable and significantly influenced overall process. This demonstrates that nHA can be prepared in shorter periods than those used in conventional synthesis methods such as hydrothermal, precipitation, and sol–gel. For the W/THF mixtures, the optimum time for maximum yield was 30 min. Using W/ACET and W/ETOH, the maximum yields were achieved after 45 min of irradiation. These results conclusively demonstrate the wide range of possibilities for obtaining nHA with a highly energetic source, such as high-frequency ultrasound.

### Thermal stability study by TGA

**Figure 7:**
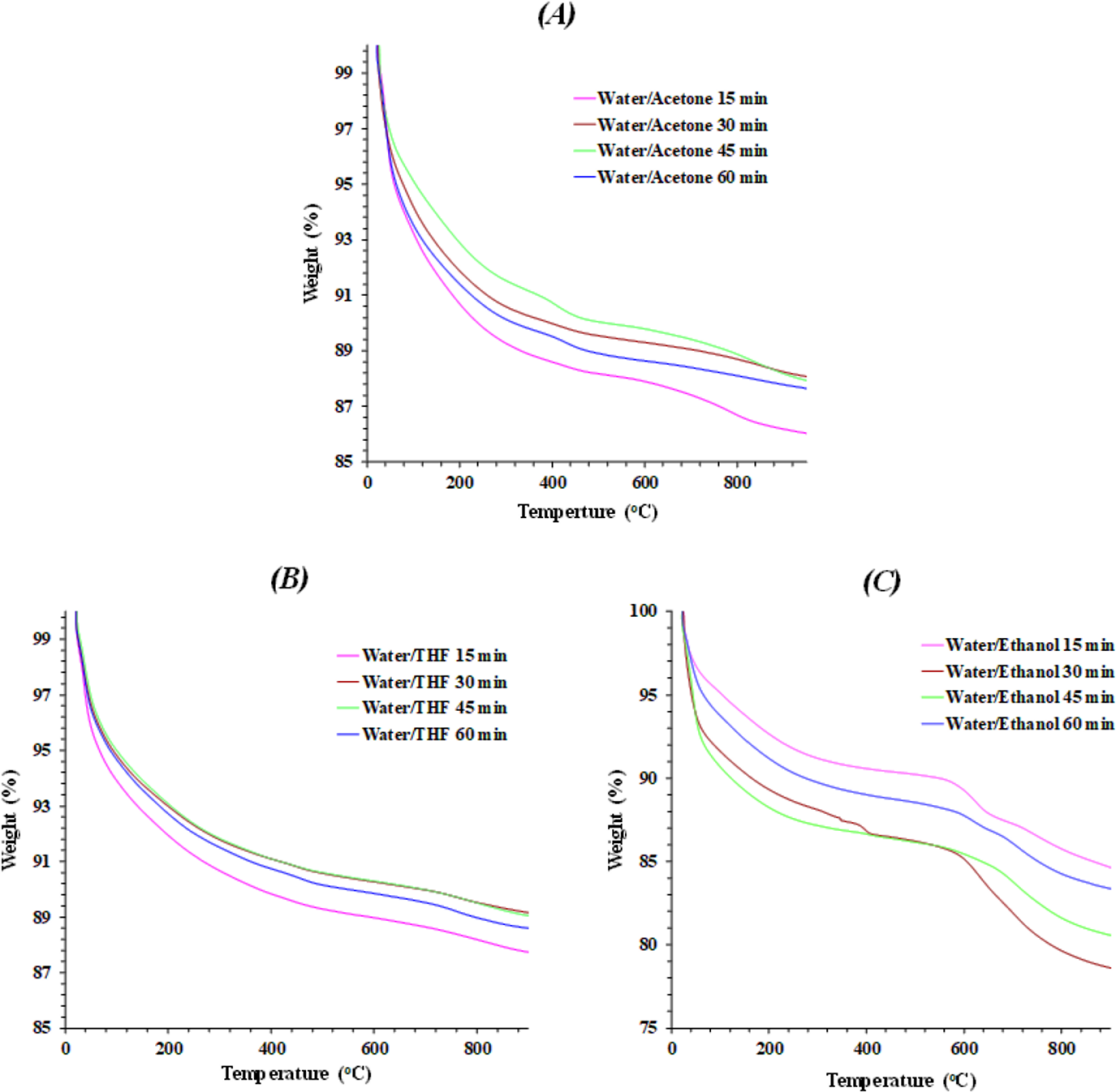
Thermograms of nHA synthesized in the mixtures: (A) W/ACET, (B) W/THF, (C) W/ETOH, at 15, 30, 45, and 60 minutes of UI.

Figure 7 shows the thermograms of the nHA samples synthesized using W/ACET, W/THF, and W/ETOH mixtures. In general, the thermal decomposition process for samples obtained with W/ACET (Figure 7A) and W/THF (Figure 7B) occurs in one single stage, with a total mass loss ranging from 11% to 15%. Almost half of this loss is attributed to the release of adsorbed water between 100 and 200 °C [68].

**W/ACET mixture:** A second, gentle slope of mass loss is likely associated with the release of structural water between 400 and 600 °C. Furthermore, the slight change in the observed slope around 400 °C for the 45 min and 60 min samples, accompanied by a mass loss ranging from 0.5% and 0.7%, could be related to the initial stages of hydroxyapatite decarbonation [69]. **W/THF mixture:** The sample that was synthesized using 15 min of ultrasonic irradiation had the highest total mass loss (12.62%). The curves for the 30 min and 45 min samples are completely overlapping, which suggests that there are no significant changes in degradation behavior. **W/ETOH mixture:** Samples synthesized in W/ETOH (Figure 7C) undergo multistage thermal decomposition, which differs from the other two mixtures. Three thermal events are clearly visible. The most notable event occurs around 600 °C for the 15 min and 60 min samples. According to FTIR and XRD characterizations, this behavior results from the formation of calcite formation as a secondary phase at these irradiation times. The small slopes that appear at 700 and 800 °C correspond to the thermal decomposition of the calcium carbonate phase [70–71], according to the following chemical reaction:


[16]

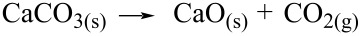



The results in Table 5 reveal a characteristic decarbonation process occurring at high temperatures. The total mass loss was significantly more pronounced in the nHA samples synthesized in the W/ETOH medium, which is consistent with the presence of calcite (CaCO_3_) previously identified by FTIR and XRD. Furthermore, a semi-quantitative phase analysis was performed to estimate the relative abundance of this secondary phase. The thermal decomposition of this secondary phase contributes to a greater release of CO_2_ compared to the nHA produced in the W/ACET and W/THF systems. In the latter cases, the total mass loss values were relatively similar and lower, corresponding to the loss of structural water plus the partial decarbonation of the hydroxyapatite lattice. These findings reinforce the conclusion that, while all systems produced carbonated nanohydroxyapatite, the W/ETOH medium uniquely promoted the co-precipitation of calcite under the studied sonochemical conditions.

**Table 5:** Mass loss percentage (adsorbed, structural, and decarbonation) of the nHA samples.

System	Irradiation time (min)	Adsorbed water (%) (room temperature to 100 °C)	Structural water (%) (200–500 °C)	Decarbonation process (%) (600–1100 °C)	Total mass loss (%)

W/ACET	15	6.63	5.09	2.14	13.86
	30	5.79	4.67	1.77	12.23
	45	4.86	5.09	2.66	12.62
	60	6.40	4.63	1.73	12.77
					
W/THF	15	5.72	4.62	2.03	12.37
	30	4.88	4.24	1.90	11.04
	45	4.80	4.40	2.07	11.29
	60	5.04	4.53	1.74	11.42
					
W/ETOH	15	4.84	4.90	6.62	16.36
	30	8.36	5.41	8.15	21.92
	45	9.18	4.54	6.10	19.83
	60	6.23	5.21	6.04	17.49

According to some reports, hydroxyapatite synthesized in W/THF and W/ACET exhibits the thermal behavior of stoichiometric hydroxyapatite [72–73]. This finding is consistent with the calcium surplus previously observed in the W/ETOH samples [74–75], and it also supports the formation of calcite. Yet, when the thermal behavior of the nHA synthesized in this study is compared with that of previous studies, it is evident that the nHA obtained here showed remarkable thermal stability [76]. It has been previously reported that hydroxyapatite only undergoes transformations leading to its decomposition at temperatures above 1000 to 1300 °C; this promotes the formation of other biocompatible phosphate species, such as tetracalcium phosphate and tricalcium phosphate [77].

### Field-emission scanning electron microscopy and particle size determination via the BET method

**Figure 8:**
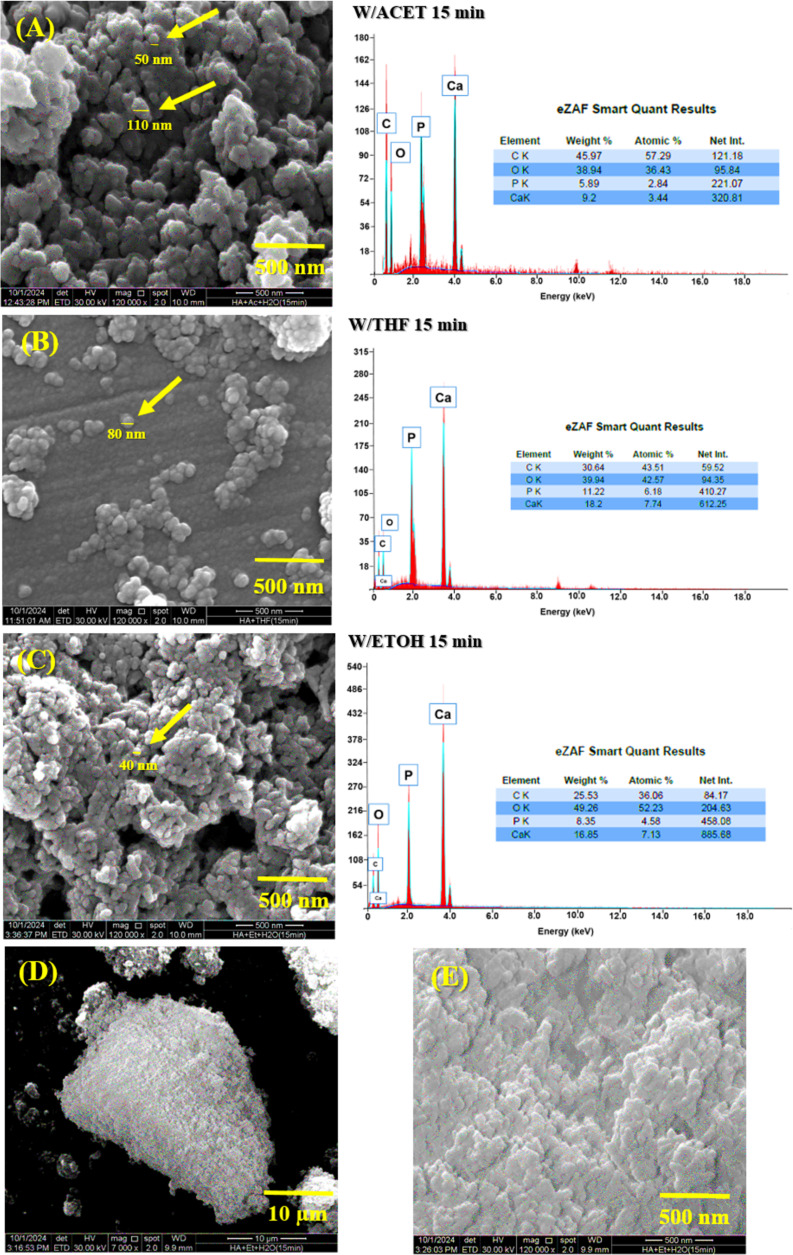
FESEM images of nHA synthesized using 15 minutes of ultrasonic irradiation: (A) W/ACET, (B) W/THF; (C–E) W/ETOH.

Figure 8 shows the morphology obtained by FESEM for the nHA obtained after 15 min of UI in the three solvent mixtures described above. **Hydroxyapatite in W/ACET:** This material consists of aggregates of spherical particles ranging in size from 60 to 70 nm. The arrangement is random, forming irregular structures (Figure 8A). **Hydroxyapatite in W/THF:** The particles are spherical, aggregated, and smaller, ranging from 50 to 60 nm in size. Its morphology resembles a cauliflower [78], with better dispersion than the W/ACET sample (Figure 8B). **Hydroxyapatite in W/ETOH:** These particles exhibit varying morphologies, including rods, spheres, and irregular particles, with a significantly smaller average size between 30 and 40 nm. FESEM also revealed for these samples a “plate or flake” morphology [79–80] (Figure 8D,E), in which particles are arranged in thin, flat, overlapping layers. This structure resembles the mineral phase found in bones [81].

The theory suggests that, in the presence of aprotic solvents in W/ACET and W/THF mixtures, which do not form hydrogen bonds, the electrostatic forces between hydroxyapatite-forming ions are robust. This results in faster nucleation, producing smaller particles with more regular morphologies. For the W/ETOH mixture, the formation of nHA with multiple morphologies and reduced crystal size was favored [82]. Additionally, the co-precipitation of secondary phases, such as calcite was observed and definitely contributed to the morphological variations. The analysis also showed that the particle size decreased with increased synthesis time in all three mixtures. This is especially relevant in terms of the yield of the synthesis reactions, as previously discussed. Details on the particle sizes obtained in each solvent mixture can be found in Section III of Supporting Information File 1.

The adsorption of a substrate onto the nanoparticle surface within a biological environment is governed by factors such as hydrophobicity, chemical structure, and particle size. In this study, this parameter is of critical importance as it directly influences the material’s performance in biological assays, such as hemocompatibility and antibacterial activity. The particle size, as discussed in the preceding paragraphs, can be determined statistically through electron microscopy imaging, as well as by calculating the specific surface area of the nanomaterial. The former method provides insights into the size associated with the boundary and geometry of particle aggregates, whereas the latter allows for an estimation of contributions from internal porosity. Here, Brunauer–Emmett–Teller (BET) analysis was employed to determine quantitative particle size distributions. The results obtained through this technique are presented below, providing a complementary perspective to the microscopic observations previously discussed. Table 6 shows the surface area values and the obtained particle sizes, in comparison to those determined by electron microscopy.

**Table 6:** Particle size for nHA obtained through BET measurements.

Sample	Irradiation time (min)	BET surface area ± SD (m^2^/g)	Particle diameter, *D*_c_ (nm)	*D*_c_ ± SD (nm)	SEM size range (nm)	SEM particle size ± SD (nm)

nHA (W/ACET)	15	98.5351 ± 0.5979	19.27	23.17 ± 5.51	60–70	65.0 ± 2.5
	60	70.1772 ± 0.2502	27.06			
						
nHA (W/THF)	15	76.2365 ± 0.4862	24.91	23.96 ± 1.35	50–60	55.0 ± 2.5
	60	82.5466 ± 0.4135	23.00			
						
nHA (W/ETOH)	15	128.8382 ± 1.5611	14.70	13.91 ± 1.18	30–40	35.0 ± 2.5
	60	145.2449 ± 1.0002	13.07			

Particle size analysis revealed a clear distinction between direct FESEM measurements and BET-derived equivalent diameters. While microscopy showed ranges between 35 and 65 nm (mean ± SD), the BET values were significantly lower: 23.17 ± 5.51 nm (W/ACET), 23.96 ± 1.35 nm (W/THF), and 13.91 ± 1.18 nm (W/ETOH). This apparent discrepancy, far from being contradictory, demonstrates a consistent correlation with the nature of the nanomaterials synthesized via sonochemistry. Such differences are typical for this synthesis method, where high surface roughness and internal porosity detected through gas adsorption, result in specific surface area diameters smaller than the geometric diameters observed in micrographs. This occurs because BET analysis integrates the total specific surface area (including contributions from internal porosity), while electron microscopy characterizes only the external geometry of the aggregates. This morphological behavior highlights the tendency of the synthesized nHA to organize into stable agglomerated structures.

In this context, the W/ETOH system exhibited the highest surface area values and the smallest particle sizes. The synthesis medium acts as a kinetic growth modulator that inhibits excessive crystal maturation and promotes the nucleation of ultrafine phases. These properties are decisive for the functionality of the biomaterial; by maximizing the density of active sites and the surface free energy, protein adsorption and chemical affinity with biological substrates are enhanced. Ultimately, this nanometric architecture, by effectively mimicking the dimensions of natural hydroxyapatite, is fundamental for optimizing cellular response and promoting tissue integration [83].

### Hemolytic effect of the nHA samples

**Figure 9:**
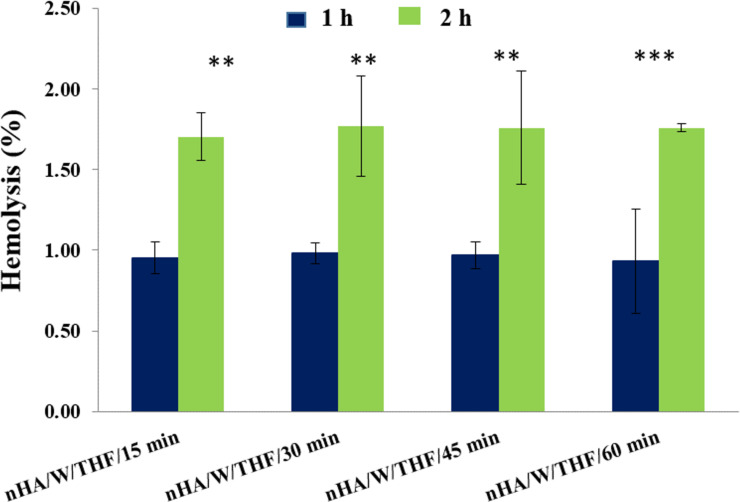
Effect of synthesized nHA in the water/THF mixture using 15, 30, 45, and 60 min of UI on the hemolysis of human blood. Values are means ± SD, *n* = 3 (three individual experiments using 4 mL of each blood donor were performed with two biological samples read as triplicates per incubation time). Two-way ANOVA and Sidak’s multiple comparisons test using GraphPad Prism 8.0.1 software were used. Differences between time points per each HA samples were considered statistically significant with *p* ≤ 0.01 (**) and *p* ≤ 0.001 (***) and Cohen’s *d* value (*d* = (*x*_2h_ – *x*_1h_)/*s*): 5 min **p = 0.0022 and *d* = 6.073; 30 min **p = 0.0015 and *d* = 3.493; 45 min **p = 0.0014 and *d* = 3.185; 60 min ***p = 0.0009 and *d* = 3.632.

Figure 9 shows the extent of the hemolytic effects of nHA samples synthesized at various UI time in W/THF solvent system. This solvent medium was selected primarily because it yielded high-purity nHA without the presence of secondary phases, such as calcite, which were observed in other media like W/ETOH. By choosing the nHA obtained from this specific system, the objective was to establish a clear baseline for the biological response of sonochemically synthesized hydroxyapatite, thereby avoiding confounding effects that secondary phases might exert on the hemolytic response. Furthermore, among the solvent systems that produced pure nHA, the W/THF samples were specifically chosen for hemolysis testing because they exhibited intermediate values for both particle size and surface area. Consequently, this sample was considered the most representative model to validate the non-hemolytic nature of the nHA produced via our ultrasonic irradiation method. These nHA samples exhibited a relatively regular morphology and demonstrated enhanced thermal stability, as evidenced by TGA studies.

As shown in Figure 9, all samples with a concentration of 1.66 mg/mL induced very low levels of hemolysis, (less than 1% for the first hour and less than 2% for the second hour of incubation), as determined using Equation 17 (Experimental section, Hemocompatibility assay subsection). There were no significant differences between the nHA samples. However, there were significant differences between the times evaluated (1 h and 2 h) for each sample. These results indicate that the synthesized nHA exhibits a high potential to be classified as non-hemolytic according to the ASTM standard F756-00 [84], which defines three levels of material hemolytic potential, namely, non-hemolytic (<2% hemolysis), slightly or mildly hemolytic (2–5% hemolysis), and highly hemolytic (>5% hemolysis). These hemolysis values are comparable to those of other hydroxyapatites. For example, Barbosa et al. [85] using washed mouse red blood cells reported less than 2% hemolysis for hydroxyapatite tested up to 1 mg/mL for 1 h; and Radha et al. [86] using washed human red blood cells reported less than 2% of hemolysis for nHA up to 40 mg/mL for 1 h and less than 4% hemolysis for nHA up to 100 mg/mL for 1 h. As the hemolytic activity is directly proportional to the diameter of the nanoparticles [86], the observed behavior could be related to the fact that the nHA obtained in the W/THF mixture exhibited very small particle sizes. At this point, it is important to highlight that the hemolytic response can be altered by the presence of any additive or by remnants of the substances used as precursors of the biomaterial, or through surface roughness. These factors can cause substantial changes in blood behavior, leading to erroneous results [87]. In this sense, the sonochemical synthesis used to obtain nHA in this research, is simple and chemically clean, aligning with the principles of green chemistry.

**Figure 10:**
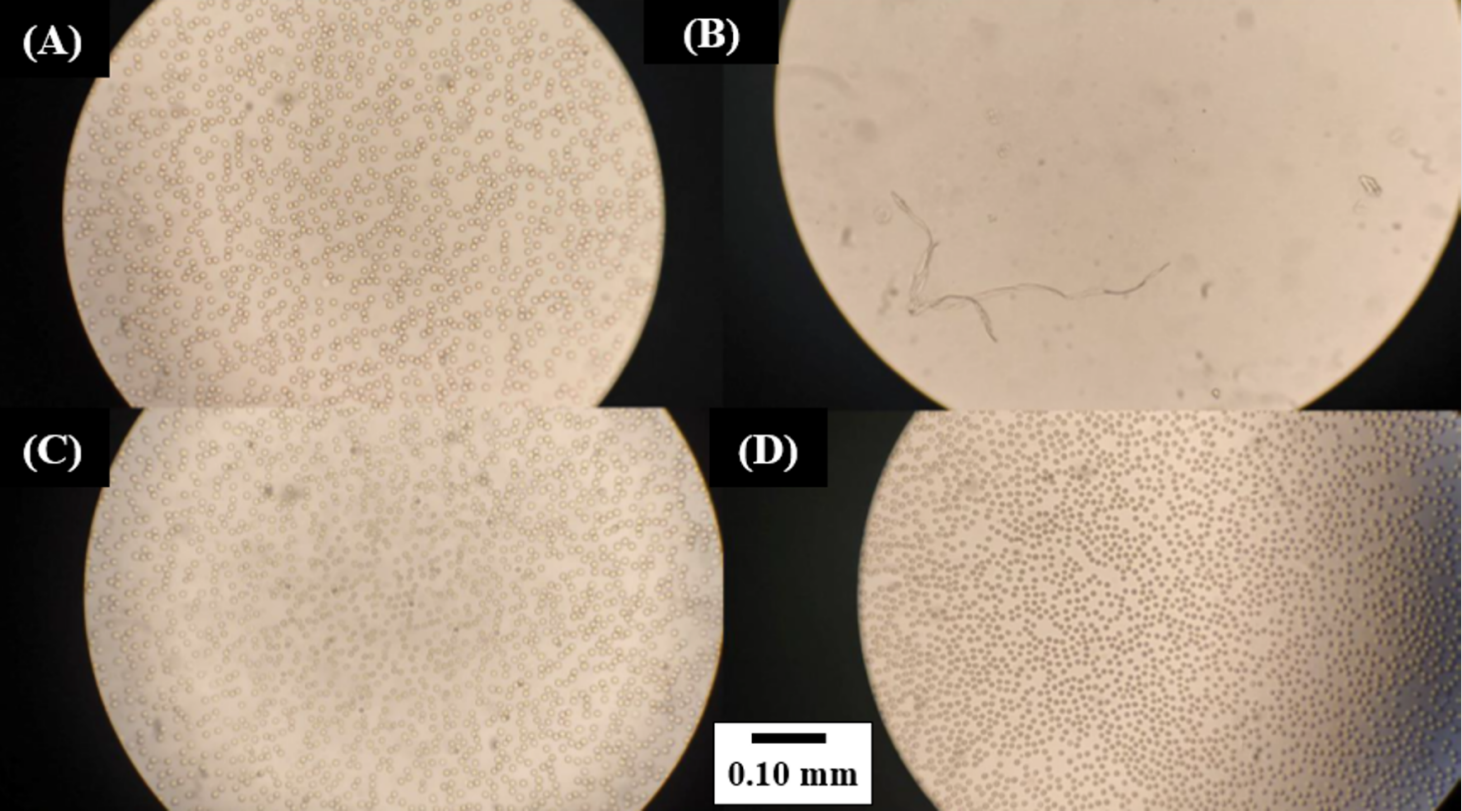
Optical microscopy images (40× magnification) of red blood cells obtained after incubation at 37 °C for 1 h with (A) 0.9% saline solution (negative hemolysis control), (B) distilled water (positive hemolysis control), and (C, D) nHA W/THF obtained using 15 and 60 minutes of UI respectively.

Figure 10 shows optical microscopy images of red blood cells from the bottom of the remaining fraction from which the supernatant was taken to test for hemolytic activity. As expected, the negative (A) and positive (B) controls show the presence and absence of red blood cells in saline solution and distilled water, respectively.

In Figure 10C, a high number of red blood cells is evident, though not quantified. These cells appear to maintain their general integrity, characterized by rounded cell contours and a clear interior, similar to those observed in Figure 10A. However, a few dark blood cells and dark external areas are also observed. In Figure 10D, slightly smaller red blood cells with a darker interior than those observed in the control (Figure 10A) are evident, though they exhibit a similar round profile. Further specialized studies are needed to determine the exact biochemical, metabolic, or osmotic nature of the darkness induced by nHA in red blood cells. A slight variation in the apparent density of red blood cells was observed when the negative control was compared to the nHA samples synthesized using 15 and 60 min of irradiation (Figure 10C,D), with the most noticeable optical contrast observed in Figure 10D. In contrast, Figure 10C,D suggest that no gross structural disruptions occurred in the erythrocytes under these conditions, as they predominantly maintain their spherical morphology. While these optical features require further investigation to rule out subtle membrane interactions, the overall maintenance of the spherical shape, combined with the low hemolysis percentages discussed previously, supports the highly promising and non-disruptive preliminary profile of the synthesized nanostructures.

When assessing the hemotoxicity of a biomaterial, platelet aggregation must also be studied. This process is crucial for preventing many circulation-related diseases and pathologies, such as strokes and heart attacks, due to its great complexity [88]. In vitro evaluation of biomaterial hemocompatibility requires studying protein adsorption, blood cell attachment (red and white cells and also platelets), and thrombus and fibrin network generation [89]. Interaction of the biomaterial with platelets must be studied since they participate in the first events that lead to the formation of blood clots, which are associated with the development of strokes and heart attacks [90]. Therefore, to evaluate the effect of nHA synthesized in W/THF on platelets, in vitro platelet aggregation tests were performed. Table 7 shows that W/THF nHA-15 and nHA-60 (nHA synthesized using 15 and 60 min of ultrasonic irradiation, respectively) at (≈0.11 mg/mL) do not affect the aggregation of human platelets. These compounds do not affect the magnitude of the maximum response (MaxA), the speed of the first phase of aggregation (SLP), nor the integral measurement of the entire aggregation process (AUC).

**Table 7:** Effect of nHA-W/THF (15 and 60 minutes) on human platelet aggregation induced by ADP.

Sample	Platelet aggregation		
	MaxA	SLP	AUC

saline solution	93.67 ± 1.44 (100%)	121 ± 18.38 (100%)	557.72 ± 18.76 (100%)
nHA-15	97.17 ± 2.31 (103.74%)	126.5 ± 11.30 (104.55%)	578.58 ± 20.83 (103.74%)
nHA-60	93.67 ± 2.08 (100.1%)	108.67 ± 10.52 (89.81%)	550.38 ± 19.55 (98.69%)

^a^Human platelet aggregation induced by ADP (10 µM) was measured by maximal aggregation (MaxA), the slope of the first phase of platelet aggregation (SLP), and the area under the curve (AUC) in the absence (control using saline solution) or in the presence of samples pre-incubated at 37 °C for 15 min. Values are means ± S.D, *n* = 3 (three individual experiments using 15 mL of each blood donor were performed with two biological samples read as triplicates). One-way ANOVA and Bonferroni post hot test using GraphPad Prism 8.0.1 software were used. Differences between the responses are not considered statistically significant.

Small variations (<10%) were observed, but they were not significant with respect to the control conditions. The inability of synthesized nHA to alter the natural process of platelet aggregation is very favorable and beneficial for potential biomedical applications and leads to its consideration as a platelet-compatible biomaterial. This result is important because platelets undergo a biological activation in the presence of foreign agents in the body. This activation causes the platelets to change their shape, adhere to each other to form clumps and release molecules and substances that regulate the immune response and blood coagulation [90]. This progressive union of platelets and coagulation factors can have negative effects on a patient’s physical integrity such as the formation of thrombi, which could lead to a stroke [91]. Based on these results it can be concluded that nHA synthesized using ultrasonic irradiation as an energy source can be considered as hemocompatible materials, as defined by ISO 10993-4 [92], since they present a very low hemolytic risk and do not interfere with the initial phase of hemostasis (platelet activation).

### Antibiofilm properties of nHA samples

**Figure 11:**
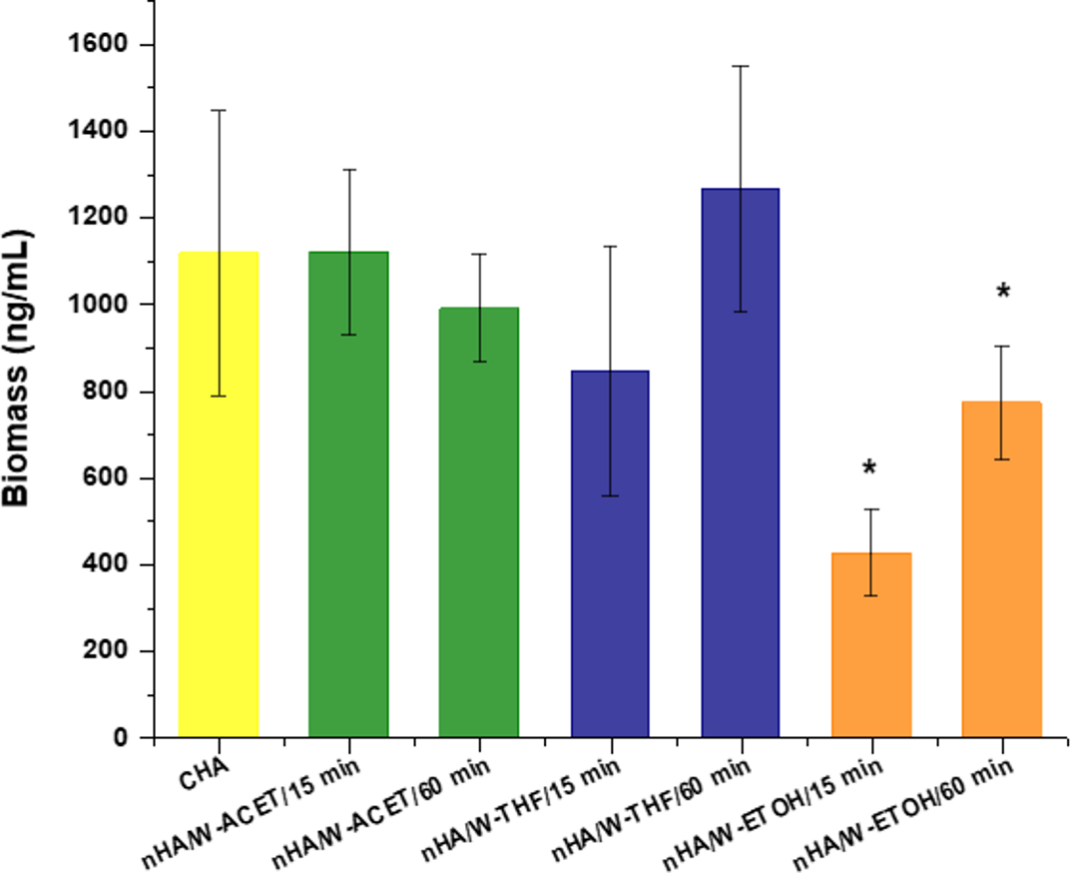
Antibiofilm potential against *P. aeruginosa* ATCC 10145 of nHA synthesized in W/ACET, W/THF, W/ETOH mixtures using 15 and 60 min of UI.

**Figure 12:**
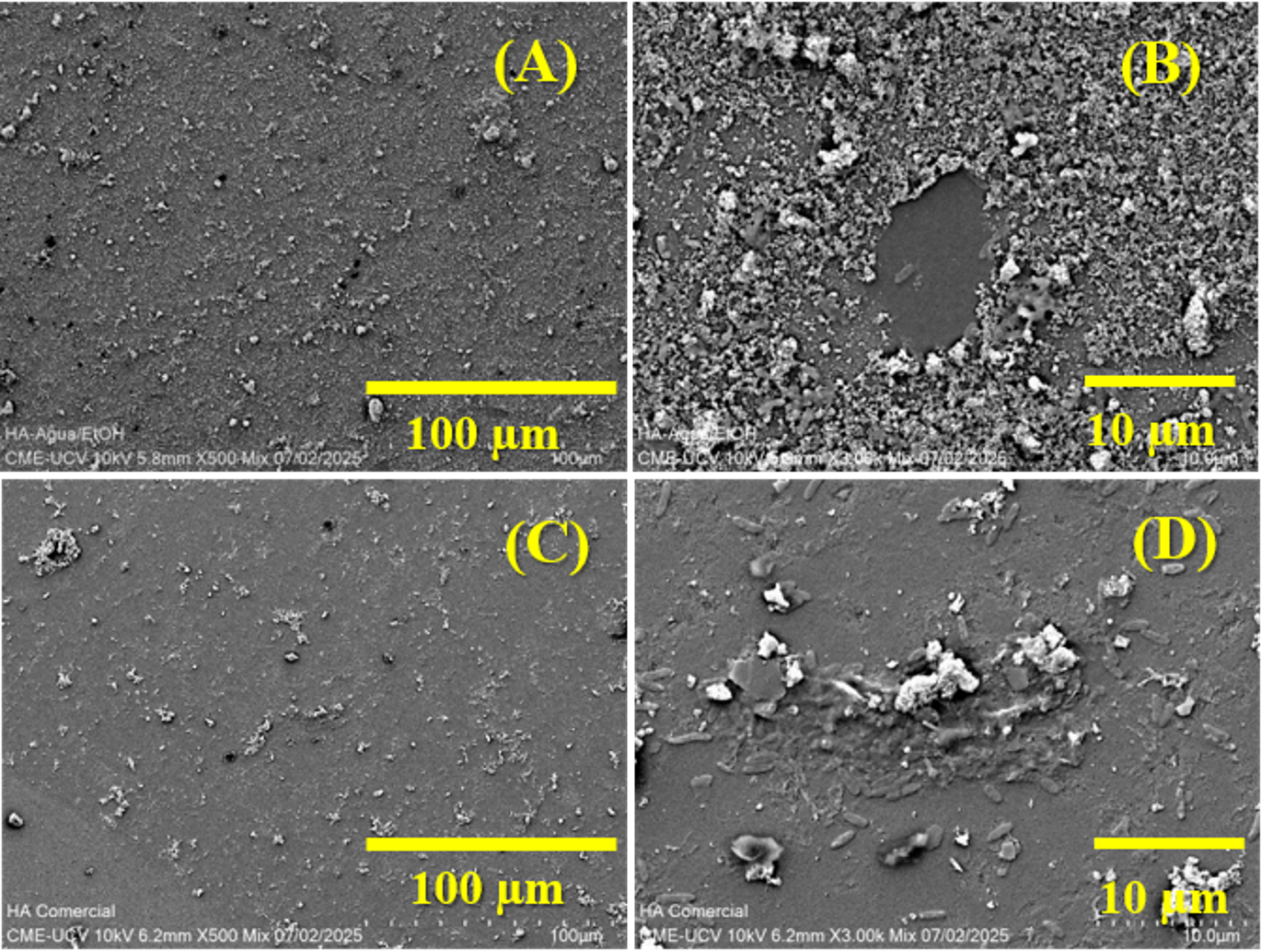
Representative SEM images of *P. aeruginosa* colonization on test surfaces of nHA variants synthesized by UI. (A, B) nHA/W/ETOH/15 min and (C, D) commercial hydroxyapatite.

This research evaluated the capability of the nHA samples to reduce the risk of *P. aeruginosa* ATCC 10145 attachment or biofilm formation, tested by crystal violet staining. This method allows for biomass quantification, which is the total mass of bacterial cell growth in a given sample. It is also a key indicator of bacterial abundance and biofilm formation. Our results showed a statistically significant reduction (*p* < 0.05) in biomass for nHA/W/ETOH/15 min (61.8%; *p* < 0.001; *d* = 2.84) and nHA/W/ETOH/60 min (30.8%; *p* = 0.007; *d* = 1.37) compared to commercial hydroxyapatite (CHA), with respective decreases of 61.8% and 30.8% (Figure 11). It is important to highlight that these enhanced antibiofilm properties are fundamentally associated with the features of the nHA phase itself (such as its smaller particle size and higher surface area). Although a secondary calcite phase was detected in these specific W/ETOH mixtures, our quantitative phase analysis using the RIR method confirmed that its content remains strictly below 6 wt %. Given this marginal and negligible concentration, any direct contribution of calcite to the biological effect can be ruled out, confirming that the structural modulation of nanohydroxyapatite is the primary driving force behind the observed biomass reduction. Additionally, SEM analysis demonstrates few rod cells (1–2 μm) adhered to the test surfaces in the nHA/W/ETOH/15 sample (Figure 12A,B). This sample was selected because it showed the highest biomass reduction. In contrast, CHA exhibited higher cellular aggregation or microcolonies embedded in exopolysaccharides with dispersed crystalline-like structures (Figure 12C,D). This phenomenon has been previously reported for biofilms of other bacterial species such as *Proteus mirabilis* [93–94]. Other highly organized structures besides crystalline-like formations, such as mushrooms, have been described in *P. aeruginosa*. These structures are believed to allow nutrients to pass from the upper to deeper levels of biofilms [95]. Finally, control surfaces exhibited no bacterial adhesion (data not shown).

Overall, this work evidences the potential use of nHA variants synthesized with UI to control *P. aeruginosa* biofilm-related infections on indwelling medical devices; it also contributes to the current knowledge of chemically friendly nHA synthesis techniques, with biomedical and antibiofilm applications. However, it is worth noting that, while the antibiofilm assays provided valuable preliminary insight, they were conducted as a single independent experiment with technical replicates. Consequently, further research with multiple independent biological replicates is needed to bolster the statistical robustness and to better understand the key mechanisms involved in the antibiofilm activity of these composites. This will allow for subsequent advancement in the prevention and control measures of bacterial colonization on hydroxyapatite prostheses and minimize public health risks.

## Conclusion

The synthesis of carbonated nanohydroxyapatite (nHA) via high-frequency ultrasonic irradiation was demonstrated, yielding a high-purity hexagonal crystalline phase without the requirement of thermal treatments. This approach aligns with the principles of green chemistry by employing a highly energy-efficient technology that uses aqueous mixtures as the reaction medium, thereby reducing the use of toxic reagents, simplifying purification stages, and establishing a sustainable synthesis process. Although the method was effective across all three evaluated systems (W/ACET, W/THF, and W/ETOH), the W/ETOH system stood out as the most efficient kinetic modulator, reaching surface areas of up to 145 m^2^/g. This architecture of ultrafine phases (14–24 nm), high internal porosity, and surface roughness confirms the capacity of ultrasound to promote nucleation and precisely modulate crystal growth.

Biological evaluation revealed excellent hemocompatibility of the material. Assays classified the nHA obtained from the W/THF system as a non-hemolytic (hemolysis < 2% at 2 h) and platelet-compatible material as it did not alter the natural process of human platelet aggregation. Furthermore, the nHA synthesized in the W/ETOH system demonstrated functional superiority over commercial hydroxyapatite, achieving a statistically significant reduction in *P. aeruginosa* biomass of up to 61.8%. Together, structural mimicry, integral biocompatibility, and antibiofilm capacity position these nanosystems as ideal candidates for tissue regeneration applications and site-specific delivery systems.

Despite these promising results, the need for exhaustive biological validation through in vitro cytotoxicity assays with osteoblastic cell lines is recognized as a limitation of this work. Future research will focus on deepening hemolysis studies and evaluating the metabolic activity and proliferative capacity of bone precursor cells in contact with these nanostructures to confirm their long-term clinical viability in tissue engineering. Furthermore, more comprehensive studies are required to determine the concentrations of Ca^2+^ and PO_4_^3−^ ions, as well as the pH effect, in order to elucidate the specific interaction mechanisms between the nHA variants (15 and 60 min) and bacterial cells.

## Experimental

### Synthesis of nanohydroxyapatite

Ca(OH)_2_ (M_W_: 74.10 g/mol, 96% purity) and (NH_4_)_2_HPO_4_ (M_W_: 132.05 g/mol, 99% purity) were used as the initial reactants to obtain nHA. The masses of these precursors were chosen to achieve a Ca/P ratio of 1.67 [96], based on the stoichiometry of the chemical reactions in Equation 18.


[18]

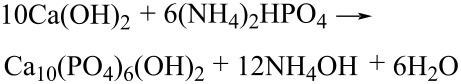



Both precursors were added to a beaker together with 40 mL of the solvent mixture, that is, W/ACET (acetone by Riedel-de Haën, bp: 66 °C, M_W_: 72.11 g/mol,), W/THF (THF by Riedel-de Haën, bp: 56 °C, M_W_: 58.08 g/mol), or W/ETOH (ethanol by Riedel-de Haën, bp: 78 °C, 46.06 g/mol); in a 1:1 ratio. The solids (calcium hydroxide and diammonium hydrogen phosphate) were dispersed in the solvents by magnetic stirring for 2–5 min. The reaction mixtures were placed in the reactor, which was subsequently sealed and purged with a nitrogen stream to displace any residual gases and ensure an inert atmosphere. The solution temperature was recorded immediately before sealing the reactor and upon completion of the ultrasonic irradiation to account for the thermal effects of cavitation. Subsequently, the mixtures were exposed to ultrasonic irradiation (UI) for controlled periods of time (15, 30, 45, and 60 min) using a Fisher 150 W Ultrasonic Generator, operating at a nominal frequency of 20 kHz. After each reaction, the resulting solid was washed several times with abundant preheated distilled water (50–80 °C), using a Thermo Scientific Sorvall Legend T+ centrifuge at 3700 rpm, in order to remove the reaction by-products. Then, the obtained nHA was dried in a Fischer Isotemp Oven 350 stove at a temperature of 40 °C and atmospheric pressure until it reached a constant weight. The dried samples were crushed in an agate mortar to achieve a fine powder consistency. All syntheses were carried out in duplicate. Table 8 summarizes the general reaction conditions.

**Table 8:** Matrix of synthesis conditions to obtain hydroxyapatite.

Experiment	Solvent mixture	Irradiation time (min)	*T*_intial_ (°C)	*T*_final_ (°C)

1	W/ACET	15	25	52
2		30	22	58
3		45	22	64
4		60	21	73

5	W/THF	15	22	49
6		30	22	52
7		45	22	68
8		60	22	62

9	W/ETOH	15	23	50
10		30	23	65
11		45	23	69
12		60	25	62

### Physicochemical and structural characterization

#### Infrared spectroscopy

Mixtures of nHA and KBr were prepared at a ratio of 200 mg KBr/0.8 mg of the sample to be analyzed. They were pulverized and thoroughly mixed in an agate mortar. Then, they were pressed at 30 tons using a press to obtain compact pellets. Finally, the pellets were analyzed using a Perkin Elmer Spectrum 100 spectrometer in the wavenumber range from 4000 to 450 cm^−1^.

#### X-ray diffraction

XRD studies were performed using a FRINGE Benchtop X-ray diffractometer from LanScientific, operating at 30 kV and 16 mA, with Cu Kα irradiation. A semi-quantitative phase analysis was performed on the XRD patterns for the series of samples obtained in the W/ETOH mixture, using the RIR (relative intensity ratio) method. To obtain the intensities of the selected nHA and calcite peaks, graphing and data analysis software was employed following this sequence of steps: (i) Peak fitting was performed using a Gaussian function to obtain the integrated area (*I*) of the main reflections for nHA [(211) plane] and calcite [(104) plane], which provided the best convergence (*R*^2^ > 0.99) for the experimental data. These results were used in the RIR method to determine the phase concentration. (ii) RIR values were obtained from the literature (ICDD database). (iii) To normalize the results to 100% of the mineralogical composition, the mass fractions of nanohydroxyapatite (W_nHA_) and calcite (W_cal_) present in the analyzed samples were finally calculated using Equation 19:


[19] $$W_{i}=\frac{\frac{I_{i}}{{\text{RIR}}_{i}}}{\sum_{j=1}^{n}\frac{I_{j}}{{\text{RIR}}_{j}}}$$

Here, RIR*_i_* is the relative intensity ratio value for phase *i*, *I_i_* is the net integrated intensity (area under the peak) of the main peak of phase *i*, determined by Equation 20 for a peak fitted using a Gaussian function,


[20] $$I_{i}=\phi \times H_{i}\times {\text{FWHM}}_{i},$$

with *H_i_* the peak net height, FWHM*_i_* the full width at half maximum, and the shape factor for Gaussian fitting, ϕ = 1.06447.

#### Thermogravimetric analysis

TGA studies were conducted to evaluate the thermal stability of hydroxyapatite. These tests were performed using TA Instrument SDT Q600 V7.0 Build 84 equipment. The different samples were analyzed at temperatures ranging from 25 to 1000 °C, in an air atmosphere at a heating rate of 10 °C/min.

#### Field-emission scanning electron microscopy and energy-dispersive X-ray spectroscopy

To reveal the morphology of the synthesized hydroxyapatite, an INSPECT-F50 field-emission scanning electron microscope, operating at 30 kV was used. The samples were prepared using two methods: In the first, called the dry method, the powder was uniformly dispersed directly onto a carbon tape. In the second, called the wet method, the samples were placed in an ethanol–water solution and stirred in an ultrasonic bath until a suspension was formed. Then, a drop was placed on an aluminum sample holder and dried in an oven at 40 °C. Finally, all samples were coated with gold.

#### Determination of specific surface area

Specific surface area (*S*_BET_) determinations were performed via nitrogen physisorption using a Micromeritics ASAP 2010 surface area and porosity analyzer (software V5.03H, Unit 1). This analysis was applied exclusively to the nHA samples synthesized in the W/ACET, W/THF, and W/ETOH systems, evaluating reaction times of 15 and 60 min. Prior to analysis, samples underwent thermal drying at 80 °C for 24 h, followed by vacuum degassing at 90 °C for 4 h to ensure the removal of adsorbed species. The surface area calculation was executed using the Brunauer–Emmett–Teller (BET) model, employing the linear region of the adsorption isotherms for mathematical fitting. The specific surface area was experimentally determined, and these values were subsequently used to calculate the derived particle diameter (*D*_c_ BET) according to Equation 21:


[21] $$S=\frac{6}{\rho \times D_{\text{c}}},$$

which relates the specific surface area *S* of the material, the crystallite size *D*_c_, and the theoretical density value of hydroxyapatite ρ (3.16 g/cm^3^) [97]. The particle size values were reported as the mean of the measurements with their standard deviation (±SD).

#### Determination of hydroxyapatite synthesis yield

The efficiency of the synthesis process with ultrasonic irradiation was determined based on the stoichiometry in Equation 19, using the relationship in Equation 22. Ammonium phosphate is the limiting reagent.


[22] $$\text{Yield}\left(\%\right)=\frac{\text{moles of nHA obtained}}{\text{moles of nHA theoretical}}\times 100$$

### Evaluation of the biological effects of hydroxyapatite samples

To evaluate the hemocompatibility of hydroxyapatite samples, hemolysis assay and in vitro human platelet aggregation tests were performed. Six volunteers gave their informed consent approved by the Human Bioethical Committee of the Venezuelan Institute of Scientific Research (DIR-0997/1569/2016).

#### Hemocompatibility assay

This analysis was performed only on nHA samples synthesized in W/THF at all UI times (15, 30, 45, and 60 min). The protocol used was modified from [98]. The collected blood (4 mL) from each healthy volunteer (*n* = 3) was anticoagulated with 3.2% sodium citrate in a 9:1 blood-to-citrate ratio. 1 mg of each nHA sample was dispersed in 300 µL of the anticoagulated blood and 300 µL of saline solution (0.9% sodium chloride), in duplicate. The samples were then mixed gently and incubated at 37 °C for 1 and 2 h under constant agitation (1000 rpm). For hemolysis controls, 300 µL of anticoagulated blood was diluted with either 300 µL of saline (negative control) or 300 µL of distilled water (positive control), and incubated in the same manner as the nHA samples. After the incubation period (1 and 2 h), 20 µL of each sample nHA and control were taken and diluted with 1000 µL of saline solution and then centrifuged at 3000 rpm for 8 min. Finally, the absorbance of each supernatant (250 µL) was measured (in triplicate) at a wavelength of 545 nm using a BioTek Power Wave XS2 spectrophotometer. The absorbance results were processed to determine the percentage of hemolysis, using Equation 17 (*A*_sample_: absorbance of the sample under study, *A*_negative control_: absorbance of the negative control (saline solution), and *A*_positive control_: absorbance of the positive control (distilled water)):


[17] $$\begin{array}{l} \text{Hemolysis percentage}\left(\%\right)=\\ \frac{A_{\text{sample}}-A_{\text{negative control}}}{A_{\text{positive control}}-A_{\text{negative control}}}\times 100 \end{array}$$

#### In vitro human platelet aggregation

Human platelets were obtained from blood (15 mL) of healthy volunteers (*n* = 3) who had not taken any drugs the previous two weeks. Platelet rich plasma (PRP) was prepared and used in platelet aggregation assays as previously described in [99]. Briefly, 10 µL of the nHA under study (4 mg/mL, suspended in saline solution) were added to 350 µL of PRP for 15 min at 37 °C prior to the stimulation of platelet aggregation (Chrono-Log model 700) by adenosine diphosphate (ADP, Sigma-Aldrich USA) (10 µM), under constant agitation (1000 rpm). Control ADP-platelet aggregation was performed using 10 µL of saline solution instead of the nHA samples. Three replicates of each condition were performed. Maximal aggregation (MaxA), slope of the first phase of platelet aggregation (SLP) and area under the curve (AUC), were calculated using the software application Aggrolink 8 (Chrono-Log Corporation, USA).

### Study of antibiofilm properties

#### In vitro biofilm assays

The *P. aeruginosa* ATCC 10145 reference strain was cultured in nutrient broth and cetrimide agar (Difco, Detroit, MI, USA) at 37 °C for 24–48 h and then stored at −80 °C. To evaluate the antibiofilm effectiveness of nHA synthesized with UI against this pathogen, one demonstrative experiment was performed by culturing the bacterial cells at early logarithmic phase (0.2 optical density) in triplicate 24-well microplates containing Luria Bertani (LB) broth, glass surfaces (1 cm^2^) with depositions of commercially available medical-grade HA (reference material), as well as nHA variants obtained with three solvent mixtures (W/ACET, W/THF and W/ETOH). The plates were incubated at 37 °C for 24 h. Prior, dispersions of all nHA samples were prepared at a concentration of 1 mg/mL. This was done by adding 5 mg of nHA to 5 mL of a 70–30% ethanol–water solution and digesting the mixture in an ultrasonic bath (42 kHz; 10 min) until it was homogeneous. When homogenization was achieved, 100 µL of the nHA suspensions were deposited onto five glass surfaces for each case and sterilized by irradiating them with a ^60^Co gamma ray source (36 kGy; JS-9500 irradiator, MDS Nordion, Canada) at the PEGAMMA Plant of IVIC. Wells containing surfaces and nHA variants without inoculum were included as negative controls. Wells containing glass surfaces and inoculum without nHA were considered as control of biofilm growth. After each incubation time, all samples were removed and rinsed three times with phosphate-buffered saline (0.1 M, pH 7.4). Then, they were transferred to wells of a microtiter dish. To quantify the biomass of the biofilms, the cells attached to the surfaces were stained with a 0.5% solution of crystal violet (CV), incubated at room temperature for 15 min and then resuspended in 95% ethanol. The absorbance at 600 nm of the resuspended CV was determined and normalized to the OD 600 nm of the corresponding grows cells density. The biomass was expressed as ng/mL [100].

To observe the morphology of the biofilms using SEM, duplicate samples were washed twice with sterile distilled water and fixed with 2.5% glutaraldehyde at 4 °C. Then, the samples were washed with cold distilled water, dehydrated with 50% ethanol for 10 min, dried at 40 °C, and coated with gold (glued to the holders using die-cut carbon conductive adhesive discs; SPI Supplies/Structure Probe, Inc., West Chester, PA, USA). Three to five different measurement positions on the surface of each sample were chosen randomly to obtain representative images of biofilm formation using a Hitachi TM 4000 (Hitachi, Tokyo, Japan) operated at a variable pressure (6–8 mm Hg) and 15 kV of acceleration voltage.

#### Statistical analysis of hemocompatibility assays

Values are expressed as the mean and standard deviation (SD), and *n* represents the number of experiments performed. Statistical analysis was performed applying one-way ANOVA and Bonferroni post hoc tests or two-way ANOVA and Sidak’s multiple comparisons test, as indicated, using GraphPad Prism 8.0.1 software. Differences between responses were considered statistically significant when the *p*-values were ≤0.05 (*), ≤0.01 (**), and ≤0.001 (***), and Cohen’s *d* value (*d* = (*x*_1_ − *x*_2_)/*s*) was calculated when two mean group resulted statistically different.

#### Statistical analysis of in vitro biofilm assays

The statistical comparisons in the biomass production among the tested surfaces were obtained using IBM SPSS Statistics version 27. Data were first screened for normality using Q–Q plots and Shapiro–Wilk test, confirming approximate normal distribution in all groups. Homogeneity of variances was assessed with Levene’s test. Due to significant heteroscedasticity (*p* = 0.005), group means were compared using Welch’s robust ANOVA followed by Games–Howell post-hoc tests for pairwise comparisons. Additionally, Dunnett’s two-tailed *t*-test was used to compare each experimental treatment against the commercial control. Effect sizes for key comparisons were calculated as Cohen’s d value using pooled standard deviations. A significance level of α = 0.05 was adopted for all tests. Observed power for the primary contrasts exceeded 0.80. All tests were two-sided. Differences between responses were considered statistically significant when the *p* < 0.05 (*).

## Supporting Information

File 1 Additional experimental data.

## Data Availability

All data that supports the findings of this study is available in the published article and/or the supporting information of this article.
