# Supplementary material for: Ultrasonic irradiation in the synthesis of nanohydroxyapatite: a chemically friendly technique for improving hemocompatibility and antibiofilm applications
Source: Beilstein J Nanotechnol. 2026 Jul 29;17:991–1015. doi: 10.3762/bjnano.17.68 (PMC13430526; doi:10.3762/bjnano.17.68)
Supplement: File 1 — Additional experimental data. [file Beilstein_J_Nanotechnol-17-991-s001.pdf]

BEILSTEIN JOURNAL OF NANOTECHNOLOGY

Supporting Information

for

Ultrasonic irradiation in the synthesis of nanohydroxyapatite: a chemically friendly technique for improving hemocompatibility and antibiofilm applications

Juan Mendoza Turmero, Cristina Parra Pantoja, Marcos Sabino Gutiérrez, Milagro Fernández-Delgado, Claudia Alvarado-Castillo, Damarys Soto Gil, María E. Gomes Gomes, Yony Gutiérrez Barrios and Daniel Suárez Arteaga

Beilstein J. Nanotechnol. **2026,** *17,* 991–1015. doi:10.3762/bjnano.17.68

Additional experimental data

License and Terms: This is a supporting information file under the terms of the Creative Commons Attribution License (https://creativecommons.org/licenses/by/4.0). Please note that the reuse, redistribution and reproduction in particular requires that the author(s) and source are credited and that individual graphics may be subject to special legal provisions.
The license is subject to the *Beilstein Journal of Nanotechnology* terms and conditions: (https://www.beilstein-journals.org/bjnano/terms)

SECTION I

Table S1: FTIR band assignment of nHA samples.

Functional group / Material		OH^- strech	C=O strech	ν_3 CO_3^{-2} (A)	ν_3 CO_3^{-2} (AB)	ν_3 CO_3^{-2} (B)	ν_3 PO_4^{-3}	ν_1 PO_4^{-3}	ν_2 CO_3^{-2} (A) and HPO_4^{-2}	ν_L OH^-	ν_4 PO_4^{-3}	ν_2 PO_4^{-3}
Commercial HA		3572	-	-	-	-	1072<br>1047	962	-	632	602<br>572	474
HA (Water/Acetone)	15 min	3569	1703	1497	1454	1423	1092<br>1040	963	875	631	603<br>567	474
	30 min	3564	-	1497	1455	1421	1088<br>1043	963	875	631	603<br>567	472
	45 min	3558	-	1499	1460	1424	1093<br>1039	963	875	631	603<br>566	472
	60 min	3564	1701	1493	1454	1423	1091<br>1038	963	875	633	603<br>565	474
HA (Water/THF)	15 min	3569	1739	1499	1458	1424	1093<br>1038	962	875	633	603<br>565	474
	30 min	3570	1737	1497	1457	1420	1097<br>1038	962	875	628	603<br>565	476
	45 min	3573	-	1495	1456	1421	1095<br>1039	962	874	630	603<br>565	476
	60 min	3561	-	1493	1457	1424	1093<br>1039	962	875	632	603<br>565	476
HA (Water/Ethanol)	15 min	3573	1712	-	1471	1424	1090<br>1036	962	873	633	603<br>565	471
	30 min	3570	-	-	1471	1420	1091<br>1039	962	875	631	603<br>566	472
	45 min	3568	-	-	1472	1420	1091<br>1036	962	874	633	602<br>565	471
	60 min	3569	1716	-	1473	1421	1093<br>1039	963	874	-	603<br>566	471

Section II. XRD patterns processed via OriginPro 2015 (64-bit) for the phase composition determination of samples synthesized in the W/EtOH system.

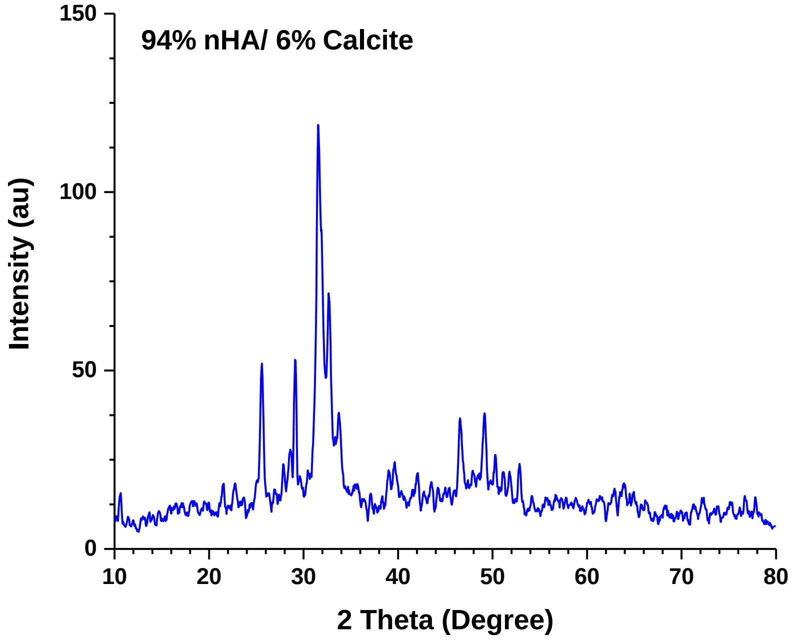

Figure S1: XRD pattern of nHA obtained in W/ETOH at 15 min of ultrasonic irradiation.

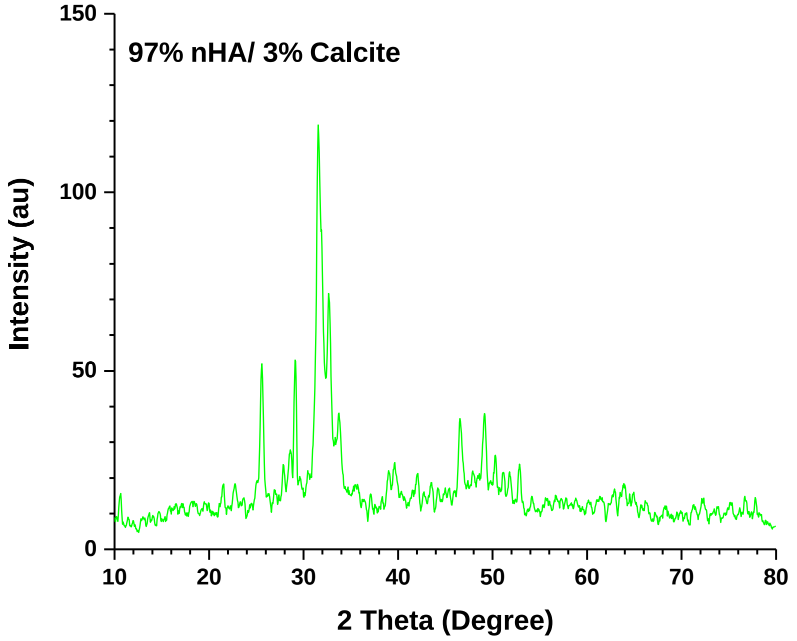

Figure S2: XRD pattern of nHA obtained in W/ETOH at 60 min of ultrasonic irradiation.

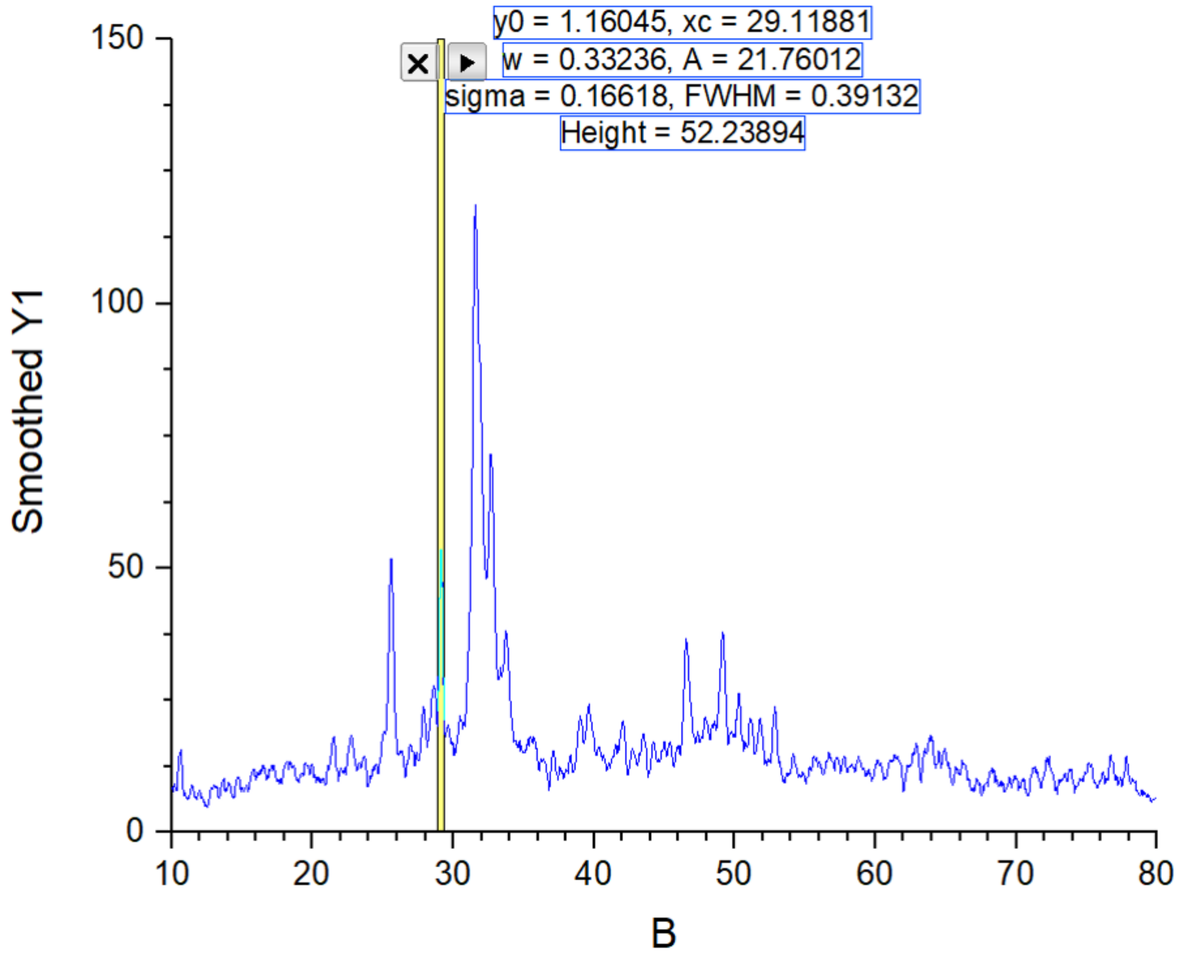

Figure S3: Calcite phase percentage quantification via signal processing of the (104) reflection using OriginPro 2015 (15 min ultrasonic irradiation).

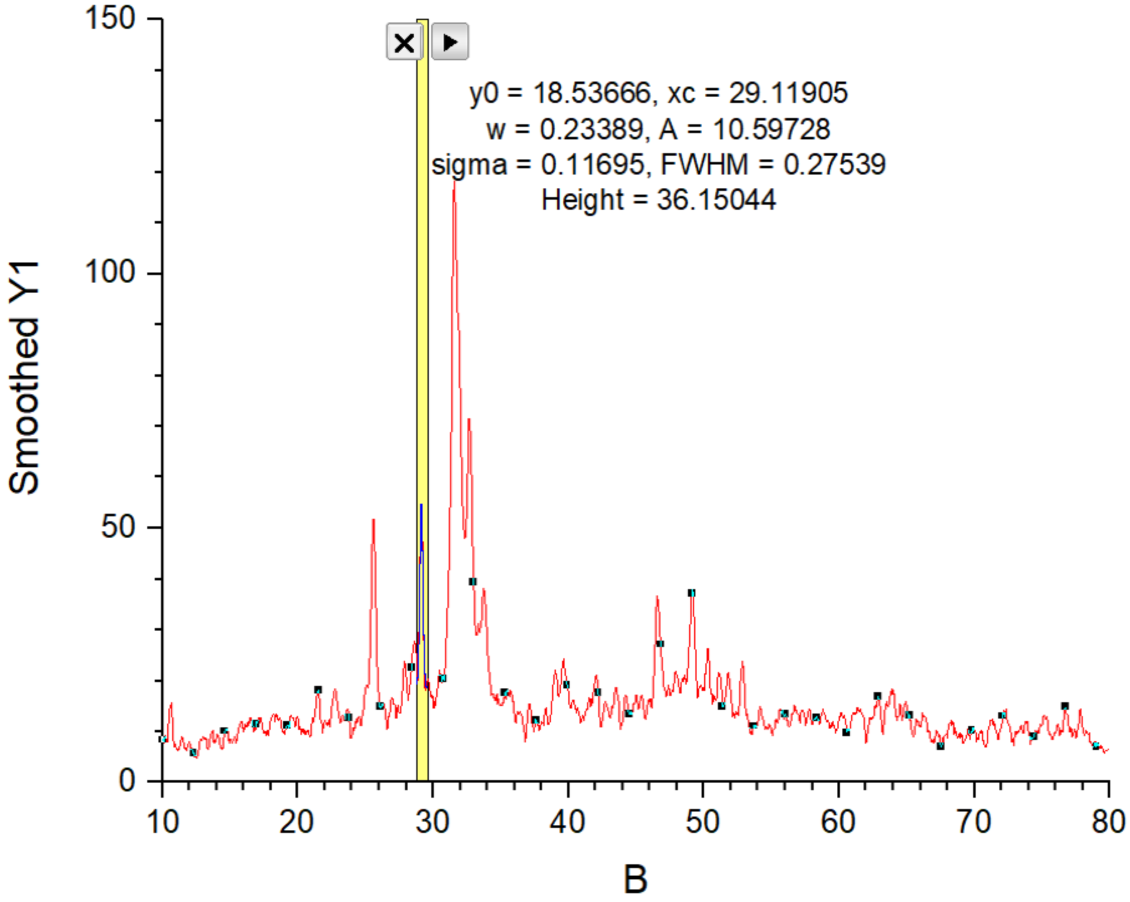

Figure S4: Calcite phase percentage quantification via signal processing of the (104) reflection using OriginPro 2015 (60 min ultrasonic irradiation).

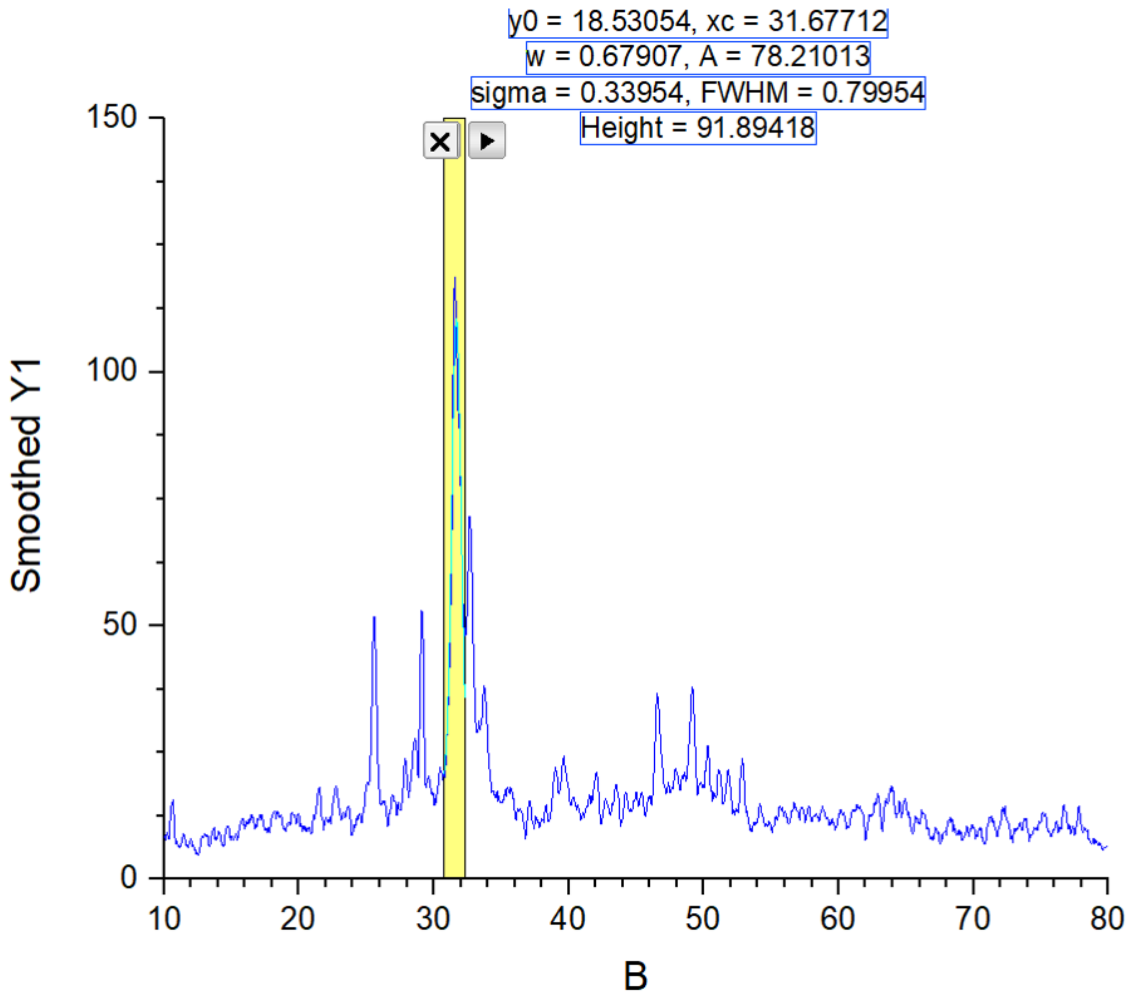

Figure S5: nHA phase percentage quantification via signal processing of the (211) reflection using OriginPro 2015 (15 min ultrasonic irradiation).

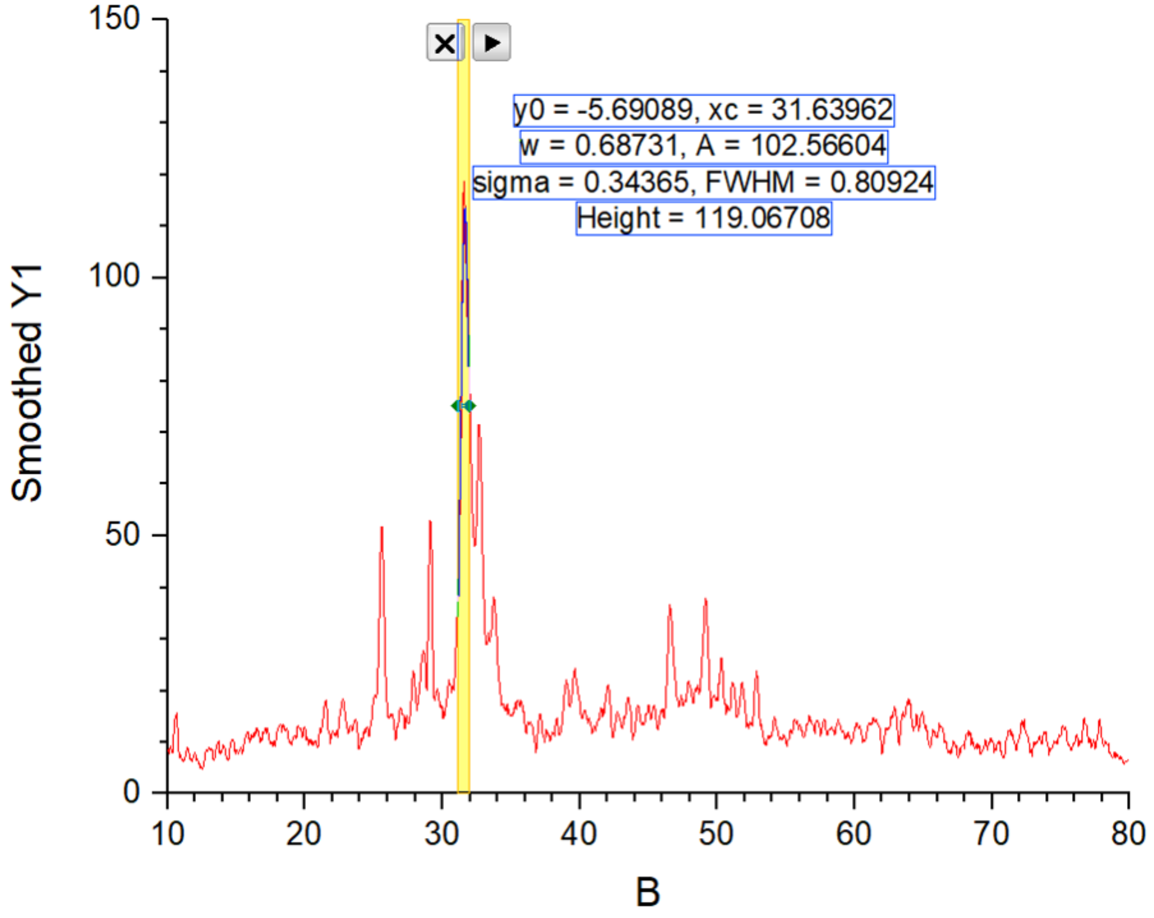

Figure S6: nHA phase percentage quantification via signal processing of the (211) reflection using OriginPro 2015 (60 min ultrasonic irradiation).

Section III. Histograms with hydroxyapatite particle sizes

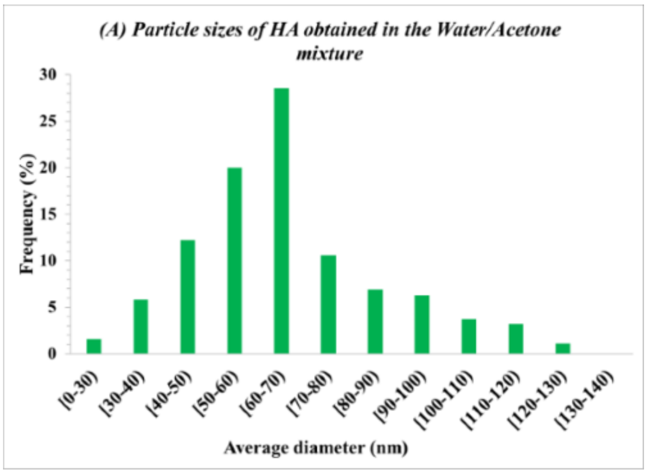

Figure S7: nHA particle sizes obtained in the W/ACET mixture at 15 minutes of UI.

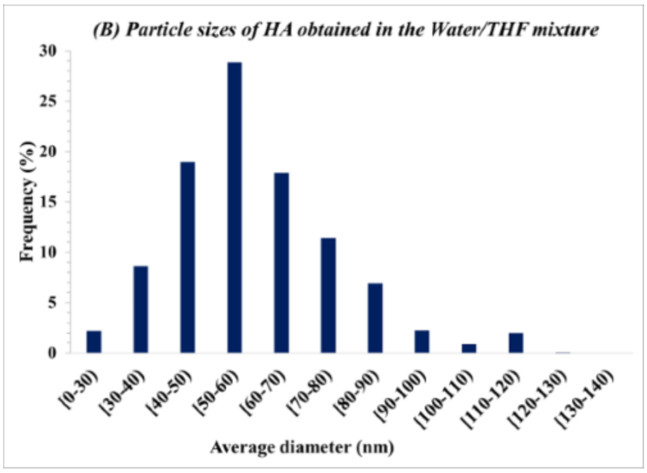

Figure S8: nHA particle sizes obtained in the W/THF mixture 15 minutes of UI.

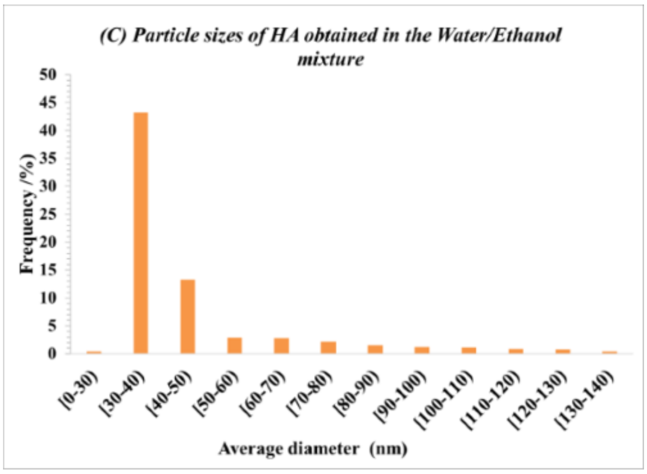

Figure S9: nHA particle sizes obtained in the W/ETOH mixture 15 minutes of UI.
